# Glycolytic potential enhanced by blockade of pyruvate influx into mitochondria sensitizes prostate cancer to detection and radiotherapy

**DOI:** 10.20892/j.issn.2095-3941.2021.0638

**Published:** 2022-08-17

**Authors:** Huan Xu, Junyi Chen, Zhi Cao, Xi Chen, Caihong Huang, Jin Ji, Yalong Xu, Junfeng Jiang, Yue Wang, Guowang Xu, Lina Zhou, Jingyi He, Xuedong Wei, Jason Boyang Wu, Zhong Wang, Shancheng Ren, Fubo Wang

**Affiliations:** 1 Department of Urology, Shanghai Changhai Hospital, Second Military Medical University, Shanghai 200433, China; 2 Department of Urology, Shanghai Ninth People’s Hospital, Shanghai 200011, China; 3 Department of Urology, the Second Affiliated Hospital of Fujian Medical University, Quanzhou 362000, China; 4 Department of Urology, No. 971 Hospital of the People’s Liberation Army Navy, Qingdao 266000, China; 5 Center for Genomic and Personalized Medicine, Guangxi Key Laboratory for Genomic and Personalized Medicine, Guangxi Collaborative Innovation Center for Genomic and Personalized Medicine, Guangxi Medical University, Nanning 530021, China; 6 Research Center of Developmental Biology, Second Military Medical University, Shanghai 200433, China; 7 Dalian Institute of Chemical Physics, Chinese Academy of Sciences, Dalian 116023, China; 8 Department of Urology, the First Affiliated Hospital of Soochow University, Suzhou 215006, China; 9 Department of Pharmaceutical Sciences, College of Pharmacy and Pharmaceutical Sciences, Washington State University, Spokane, WA 99202, USA

**Keywords:** Glycolytic potential, prostate cancer, mitochondrial pyruvate carrier (MPC), mitochondria pyruvate influx, diagnosis, radiotherapy

## Abstract

**Objective:**

This study aimed to evaluate the effects of mitochondrial pyruvate carrier (MPC) blockade on the sensitivity of detection and radiotherapy of prostate cancer (PCa).

**Methods:**

We investigated glycolysis reprogramming and MPC changes in patients with PCa by using metabolic profiling, RNA-Seq, and tissue microarrays. Transient blockade of pyruvate influx into mitochondria was observed in cellular studies to detect its different effects on prostate carcinoma cells and benign prostate cells. Xenograft mouse models were injected with an MPC inhibitor to evaluate the sensitivity of ^18^F-fluorodeoxyglucose positron emission tomography with computed tomography and radiotherapy of PCa. Furthermore, the molecular mechanism of this different effect of transient blockage towards benign prostate cells and prostate cancer cells was studied *in vitro*.

**Results:**

MPC was elevated in PCa tissue compared with benign prostate tissue, but decreased during cancer progression. The transient blockade increased PCa cell proliferation while decreasing benign prostate cell proliferation, thus increasing the sensitivity of PCa cells to ^18^F-PET/CT (SUVavg, *P* = 0.016; SUVmax, *P* = 0.03) and radiotherapy (*P* < 0.01). This differential effect of MPC on PCa and benign prostate cells was dependent on regulation by a VDAC1-MPC-mitochondrial homeostasis-glycolysis pathway.

**Conclusions:**

Blockade of pyruvate influx into mitochondria increased glycolysis levels in PCa but not in non-carcinoma prostate tissue. This transient blockage sensitized PCa to both detection and radiotherapy, thus indicating that glycolytic potential is a novel mechanism underlying PCa progression. The change in the mitochondrial pyruvate influx caused by transient MPC blockade provides a critical target for PCa diagnosis and treatment.

## Introduction

Metabolic reprogramming, one of the most critical characteristics of malignant tumors, plays an important role in cancer development and progression^1,2^. In contrast to normal cells, cancer cells often exhibit high glycolysis levels even with an abundant oxygen supply, in a phenomenon called the Warburg effect^3^. The robust increase in glycolysis stimulates cancer cell proliferation, owing to the rapid production of ATP and important metabolites, thus contributing to cancer cell survival. However, heterogeneities in cancer metabolism may be caused by cancer types and tumor stages^4,5^. Glycolysis is not significantly elevated in some cancers^6,7^, particularly prostate cancer (PCa); consequently, PCa is relatively insensitive to ^18^F-fluorodeoxyglucose-positron emission tomography (^18^F-FDG-PET)-based imaging^8,9^.

Pyruvate is the end-metabolite produced in glycolysis, which occurs in the cell matrix rather than within mitochondria. Pyruvate is produced mainly from glucose, which is used and taken up by cells from the environment. Pyruvate crosses the mitochondrial membrane, where it is converted into acetyl-CoA and enters the tricarboxylic acid (TCA) cycle for further oxidation, thereby producing ATP and energy for cell growth. The mitochondrial pyruvate carrier (MPC), a novel regulator of pyruvate uptake in the inner mitochondrial membrane, has been reported to regulate glycolysis and TCA cycle levels^10,11^. The MPC complex is formed by 2 different units, MPC1/2, and has been shown to be blocked by the small molecular compound UK5099^12,13^. MPC serves as a gateway for pyruvate entry into the mitochondria and is downregulated in most cancers exhibiting the Warburg effect^14–16^. However, PCa presents a different metabolic form in which Warburg effect is not significantly observed. MPC has not been well studied in cancers with glycolytic reprogramming similar to that in PCa. Mitochondria are the site of the TCA cycle, and mitochondrial dynamics is the most critical pathway in mitochondrial homeostasis, which is regulated by ATP levels and the mitochondrial membrane potential (MMP)^17^. ATP maintains the MMP, thus stabilizing mitochondria and allowing cells to grow and survive. MPCs are located on the inner mitochondrial membrane and may serve as important regulators of the balance between glycolysis and the TCA cycle. More work is needed to clarify the effects of MPCs on mitochondrial homeostasis.

Here, we show that transient blockade of pyruvate influx into mitochondria has opposite effects in PCa cell lines and non-carcinoma prostate cell lines, and causes a glycolytic potential (GP) phenotype in the former, in a manner potentially regulated by the VDAC1-MPC-mitochondrial homeostasis-glycolysis pathway. Transient promotion of glycolysis by MPC blockade sensitizes PCa to ^18^F-FDG-PET-computed tomography (CT) detection and radiotherapy (RT).

## Materials and methods

### Study population

#### Metabolic profiling

In total, 76 PCa and 19 benign prostatic hyperplasia cases were examined in this metabolomics study. Pairs of PCT and adjacent normal tissue from each patient with PCa were collected from the archives of the Urological Department of Shanghai Changhai Hospital. The details were as previously published^18,19^.

#### RNA-Seq data

The samples were collected as previously reported^20^. Briefly, radical prostatectomy specimens from 65 patients were included in group A, and specimens from 125 patients were included in group B. The results have been described in detail in **Supplementary Table S2** of our previous publication^20^, and a brief summary is also provided in the supplemental materials.

#### Tissue microarray

This retrospective study was performed at the Department of Urology at Shanghai Changhai Hospital and was approved by the medical ethics review committee of Shanghai Changhai Hospital (approval number: CHEC2019-110). A total of 210 patients with PCa were enrolled. Information on the tissue microarray (TMA) was as previously reported^21,22^. The H^−^ score was calculated as follows: percentage of weak staining (scale: 0 – 100) × 1 + percentage of moderate staining (scale: 0 – 100) × 2 + percentage of strong staining (scale: 0 – 100) × 3.

### Bioinformatics analysis

The genes positively associated with glycolysis were selected for gene set variation analysis (GSVA). GSVA and gene set enrichment analysis (GSEA) were performed with open-source software packages for R downloaded from http://www.bioconductor.org

### Epigenetic data

The epigenetic data for VDAC1 were downloaded from the public database Washu Epigenome Browser (http://epigenomegateway.wustl.edu/browser/). Then H3K27ac, H3K4me3, and H3K4me1 were used for the epigenetic analysis of VDAC1 in different cell lines.

### MMP measurement

We used JC-1, a fluorescent dye commonly used as an MMP probe to measure the mitochondrial membrane potential in various cell lines. At higher concentrations (in the presence of high MMP), the dye forms J-aggregates that exhibit a broad excitation spectrum and an emission maximum at ~590 nm. At low concentrations (in the presence of low MMP), JC-1 is predominantly a monomer that yields green fluorescence with emission at ~530 nm.

### Cellular ATP assays

Cellular ATP levels were measured with a luciferase-based ATP assay kit (Beyotime, S0026, China) according to the manufacturer’s instructions.

### Seahorse analysis

UK5099 treatment was performed 1 day before Seahorse analysis. The experiments were performed on a Seahorse XF24 Analyzer.

### Metabolomic profiling based on GC–MS

For cells cultured in medium containing ^13^C-glucose, a GCMS-QP 2010 Plus quadrupole mass analyzer was used for nontargeted scanning.

### Animal procedures

The nude mice were 5-week-old males (Shanghai Laboratory Animal Center, SLAC, China). A total of 5 × 10^6^ PCa cells were suspended in 0.1 mL PBS and inoculated subcutaneously into the nude mice. The tumor volume was calculated with the formula volume (mm^3^) = (length × height^2^)/2. UK5099 was administered to mice through intraperitoneal injection at a dose of 6 mg/kg BW. The time periods are shown in the figures. The animal care and experimental procedures were approved by the Institutional Animal Ethics Committee (approval No. SH9H-2021-A26-1). For CT scanning, the mice were anesthetized with isoflurane. Cervical dislocation or CO_2_ inhalation were used for euthanasia.

### RT

A ^60^Co source in the Irradiation Center (Faculty of Naval Medicine, Second Military Medical University, China) was used for irradiation as previously described^23^. All irradiated animals received a single dose of 6 Gy.

### PET-CT

The animals were fasted 6–8 h before ^18^F-FDG micro-PET/CT imaging (Super Nova^®^ PET/CT, Pingseng, China). The images were assessed with 3D segmentation with PET in Avatar 1.2 software (Pingseng, China). For the ^18^F-glucose PET/CT, we used different time points from 15 min to 150 min. Ninety minutes was the most significant time point and was chosen as the time point for use in subsequent studies.

### Statistical analysis

Statistical analysis was performed in SPSS software (version 19.0; SPSS Inc., Chicago, IL, USA), and the figures were generated in GraphPad Prism 5 (San Diego, CA, USA). Pearson correlation analysis was performed. A *P* value < 0.05 was considered significant.

### Ethics approval and consent to participate

This study was approved by the Clinical Research Ethics Committee of Shanghai Changhai Hospital (Approval No. CHEC2019-110). All clinical samples were obtained from Shanghai Changhai Hospital (Shanghai, China). Written informed consent was obtained from the participants before sampling. Animal ethical approval was provided by the Shanghai Ninth People’s Hospital with Approval No. SH9H-2021-A26-1.

## Results

### Mitochondrial pyruvate uptake contributes to PCa development, on the basis of integrated transcriptomics and metabolomics

**Figure 1:**
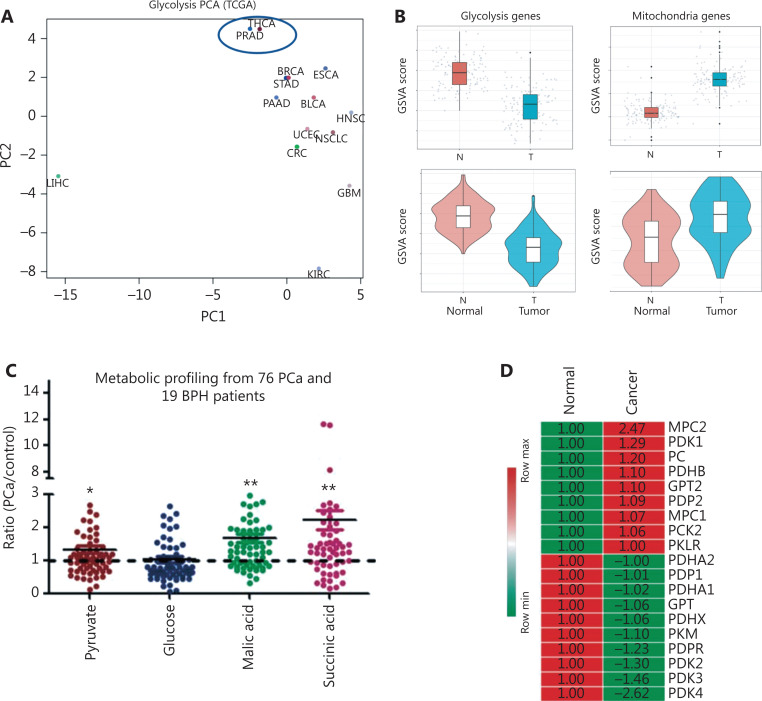

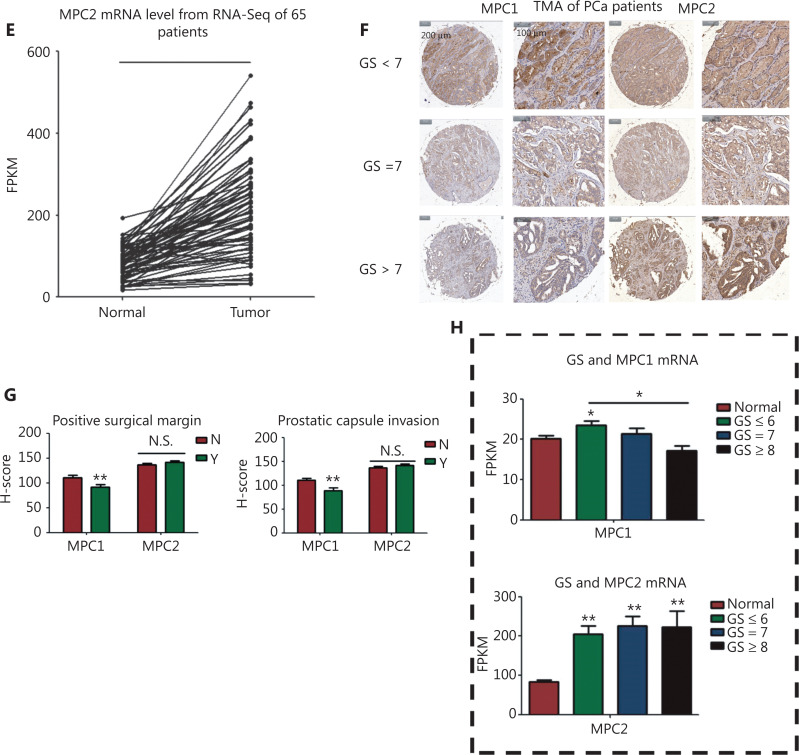
Metabolic signatures and changes in MPC expression in PCa. (A) Glycolytic characteristics of the different types of cancers. The data are from TCGA database. (B) Metabolic levels of benign prostate and PCa tissues (*n* = 65). (C) Fold changes in metabolic profiling from prostate tissues of patients with PCa. These data are based on 76 patients with PCa and 19 patients with benign prostate hyperplasia. (D) Heatmap of pyruvate-associated genes. The fold changes are marked. Red indicates an increase in PCa, whereas green indicates a decrease. (E) MPC2 expression analysis (*n* = 65). (F) TMA (*n* = 210) analysis of MPC1 and MPC2.The bars are 200 μm and 100 μm, as marked. (G) TMA (*n* = 210) analysis of MPC1 and 2 expression levels in different clinical outcomes (N = no, Y = yes). (H) Changes in MPC1 and 2 in the RNA-Seq data of PCa according to the different Gleason grades. *P < 0.05, **P < 0.01, compared with the control group.

To study glycolytic reprogramming in cancers, we selected glycolysis-associated genes (listed in **Supplementary Table S1**) for bioinformatics analysis with open access data from The Cancer Genome Atlas (TCGA) database. Principal component analysis (**Figure 1A, Supplementary Figure S1, S3 and S4**) indicated that glycolysis greatly varied with cancer type. We observed a diminished glycolysis level in PCa in our own database of paired tissues from 65 patients. Glycolysis-associated genes were dramatically downregulated in the PCTs (**Figure 1B and Supplementary Figure S1**), whereas mitochondria-associated genes (listed in **Supplementary Table S1**) were significantly upregulated (**Figure 1B**). All the data indicated that in PCa, glycolysis decreased, whereas mitochondrial activity significantly increased.

To better understand the changes in glycolysis, we performed metabolic profiling of PCa cancer and para-cancerous tissues. Accordingly, TCA-cycle-associated metabolites were significantly elevated (**Figure 1C and Supplementary Figure S1**). Pyruvate, an important bridge between the TCA cycle and glycolysis, was significantly elevated in PCT (*P* < 0.05, **Figure 1C**). We then integrated metabolic profiling with our transcriptomic data, and identified MPC2 as the most significantly upregulated gene (**Figure 1D and Supplementary Figure S1**). If MPC1 or MPC2 is downregulated, the other unit is not stable and is also downregulated^11,16^.

Prostate cancer exhibited abundant MPC2 (**Supplementary Figures S1 and S5**), which was higher in patients with PCa than in controls, according to RNA-Seq data. We further evaluated the role of MPC during PCa progression by applying our TMA to 210 patients. MPC1 was negatively associated with the Gleason score (**Figure 1F and 1H**), prostatic capsule invasion, and positive margins of surgical specimens (**Figure 1G**, MPC1, *P* < 0.01). Thus, the MPC complex plays an important role in metabolic reprogramming during PCa development and progression.

### Transient blockade of mitochondrial pyruvate uptake promotes PCa cell proliferation while inhibiting BPH-1 cell proliferation

**Figure 2:**
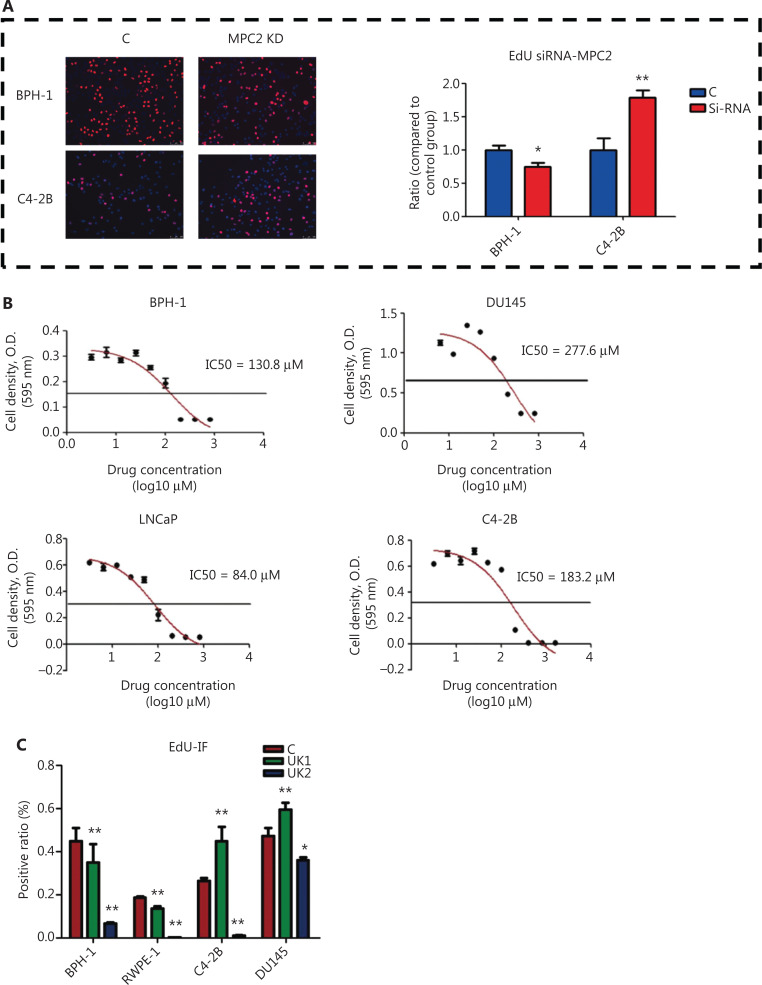

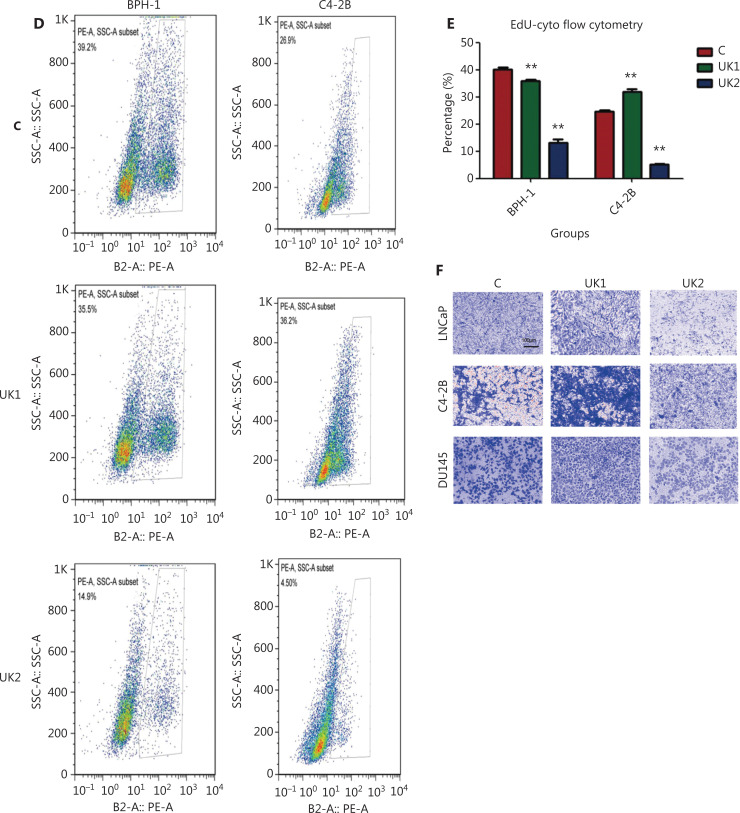
Dual effects of transient blockade of mitochondrial pyruvate influx on the proliferation of benign prostate cells and PCa cells. (A) EdU analysis of empty vector (C) and MPC2 KD cells. (B) IC50 for UK5099 in different cell lines. (C) EdU analysis of MPC inhibitor-treated cells with fluorescence staining. (D) EdU analysis of UK5099-treated cells with flow cytometry. (E) EdU analysis of the UK5099-treated cells shown in **Figure 2D**. (F) Transwell analysis of UK5099-treated cells. The data are represented as the mean ± SEM. *P < 0.05, **P < 0.01, compared with the control group; UK1: 10 μm UK5099; UK2: 100 μm UK5099; EV: empty vector; MPC: MPC1/2 overexpression. All experiments were performed more than 3 times.

To block pyruvate from entering the mitochondrial TCA cycle, we used MPC2 siRNA (knockdown efficiency shown in **Supplementary Figure S7**) and the classic MPC inhibitor UK5099 at 2 different doses (UK1, 10 μm; UK2, 100 μm) in cell culture. We assessed the half maximal inhibitory concentration (IC50) of UK5099 in PCa and BPH-1 cells (**Figure 2B**). On the basis of previous reports^15,12^ and our IC50 testing, we selected 2 different doses of UK1 and UK2 for further studies. Interestingly, after 24 h of incubation, UK1, in agreement with the results of transient downregulation of MPC, tended to improve proliferation in PCa cells (C4-2B and DU145 cell lines) while inhibiting proliferation in non-carcinoma prostate cells (BPH-1 and RWPE1 cell lines, **Figure 2C**).

Flow cytometry was used to confirm the cell cycle and proliferation (**Figure 2D and 2E**). In addition, Transwell assays indicated that low-dose MPC inhibitor treatment promoted PCa migration (**Figure 2F**). Colony formation assays similarly confirmed that transient blockade of mitochondrial pyruvate influx enhanced PCa cell line proliferation while inhibiting the BPH-1 cell line (**Supplementary Figure S7**). Thus, the transient blockade of pyruvate influx into mitochondria promoted proliferation in PCa cells while inhibiting proliferation in noncancer prostate cells.

### Transient blockade of mitochondrial pyruvate influx regulates ATP levels differently between PCa and benign prostate cells

**Figure 3:**
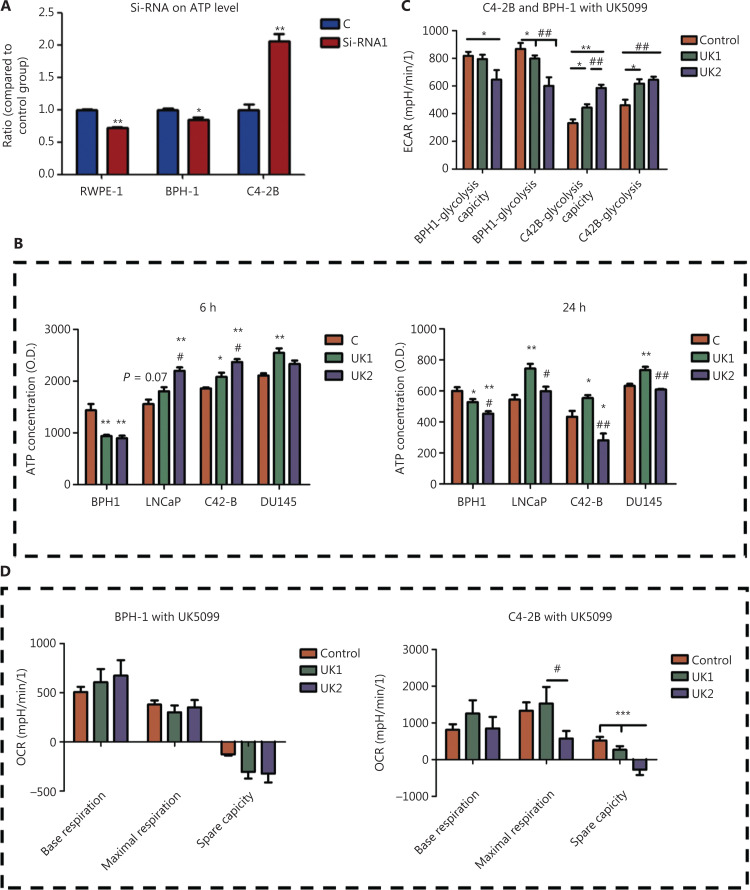

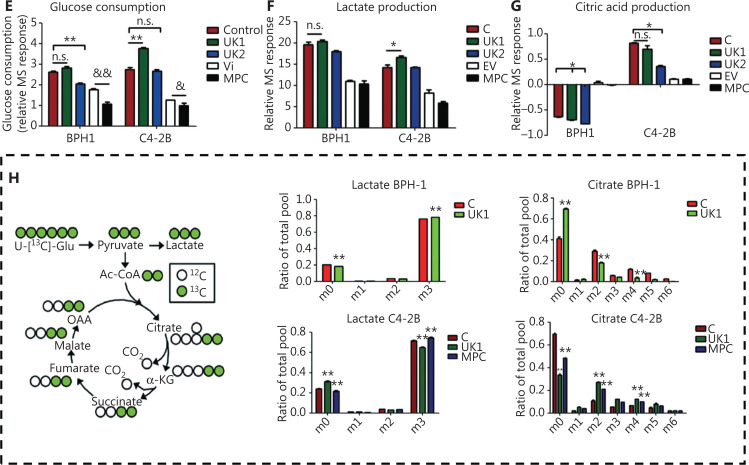
Changes in energy and metabolism after the blockade of pyruvate into mitochondria. (A) Changes in ATP after 24 h of treatment with MPC2 siRNA. (B) Changes in ATP with MPC inhibitor treatment after 6 h and 24 h. (C) Changes in ECAR after treatment with UK1 and UK2. (D) OCR changes after treatment with UK1 and UK2. (E) Glucose consumption in BPH-1 and C4-2B cell lines after MPC inhibitor treatment and MPC overexpression. (F) Lactate production in BPH-1 and C4-2B cell lines after MPC inhibitor treatment and MPC overexpression. (G) Citrate acid production in BPH-1 and C4-2B cell lines after MPC inhibitor treatment and MPC overexpression. (H) D-[U-13C] glucose tracer for detecting the contribution of glucose metabolism to the TCA cycle. The data are presented as the mean ± SEM. *P < 0.05, **P < 0.01, compared with the control group; ^#^*P* < 0.05, ^##^*P* < 0.01, compared with the UK1 group; UK1: 10 μm UK5099; UK2: 100 μm UK5099; EV: empty vector; MPC: MPC1/2 overexpression; ^&^*P* < 0.05, ^&&^*P* < 0.01, compared with the EV group. All experiments were performed more than 3 times.

The ATP levels increased significantly (*P* < 0.01) with MPC2 siRNA knockdown in the PCa cell line but decreased in BPH-1 cells (*P* < 0.05) in the first 24 h (**Figure 3A**). A low-dose MPC inhibitor increased ATP levels in the C4-2B cell line while decreasing ATP levels in the BPH-1 cell line. UK2 also elevated the PCa ATP levels (*P* < 0.01, **Figure 3B**) in the first 6 h after UK5099 treatment. Given that ATP changes are caused by the glycolysis/TCA cycle balance, we measured the extracellular acidification rate (ECAR) and oxygen consumption rate (OCR) in the BPH-1 and C4-2B cell lines (**Figure 3C and 3D**). After culturing for 24 h, C4-2B cells tended to show an enhanced glycolysis level and glycolytic capacity. However, glycolysis decreased in BPH-1 cells after 24 h of UK1 and UK2 treatment. No significant differences were observed in OCR in BPH-1 cells, whereas UK2 tended to elevate the maximal respiration level after UK1 treatment (**Figure 3D**). Together, the ATP measurements and Seahorse data showed that PCa induced a faster response and compensative effect when pyruvate transport into mitochondria was transiently blocked.

### PCa cells exhibit a stronger interaction between glycolysis and the glucose TCA cycle than benign prostate cells

To observe the metabolite changes critical for ATP production, we used mass spectrometry to detect the variation in metabolites associated with glycolysis and the TCA cycle. In agreement with the above results, UK1 accelerated glucose uptake in C4-2B cells. However, no significant difference was observed in BPH-1 cells treated with UK1 (*P* < 0.01, **Figure 3E**). Lactate production, which has been suggested to be a glycolysis marker^24^, increased in C4-2B cells after 24 h of UK1 treatment, whereas no significant changes were observed in the BPH-1 cell line (**Figure 3F**). Moreover, in the control group, the citric acid production results indicated that BPH-1 cells were more affected by the TCA cycle than C4-2B cells (**Figure 3G**). After UK5099 treatment, the TCA cycle was downregulated in both cell lines, and this effect was dose dependent, as shown in **Figure 3G**. Therefore, the transient blockade of MPC decreased glucose influx in BPH-1 cells while upregulating glucose influx in C4-2B cells.

### Transient mitochondrial pyruvate influx sensitizes PCa to ^18^F-FDG-PET-CT and RT

**Figure 4:**
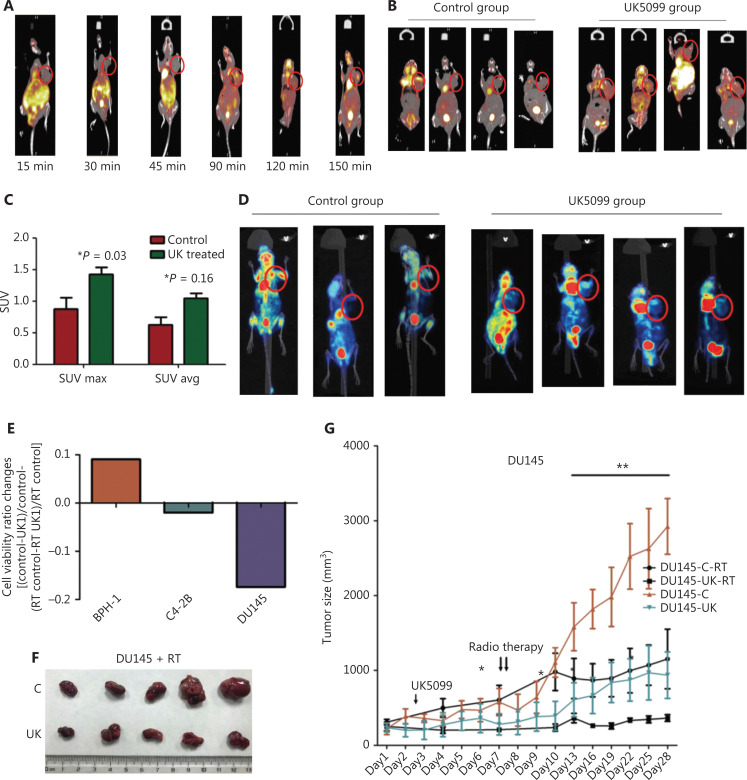

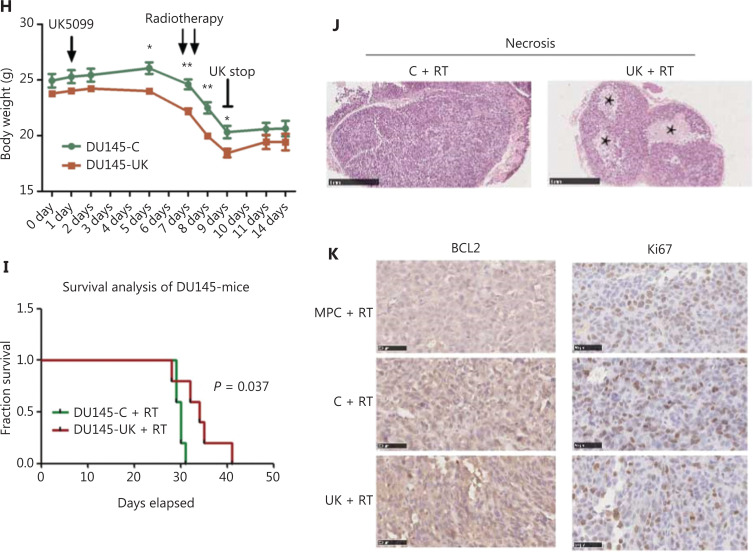
*In vivo* study of the MPC inhibitor. (A) ^18^F-FDG-PET-CT analysis at different time points. (B) ^18^F-FDG-PET-CT analysis of the tumor treated with UK5099 1.5 h after UK5099 i.p. injection. (C) SUVmax and SUVavg in mice injected with UK5099. (D) 3-D remodeling PET analysis of the tumor treated with UK5099 1.5 h after UK5099 i.p. injection. (E) RT sensitivity of PCa cells with UK5099 treatment. (F) RT sensitivity of subcutaneous PCa in mice treated with UK5099. (G) Tumor size and RT sensitivity in mice burdened with subcutaneous PCa after UK5099 treatment. (H) Weight change during the RT + UK5099 treatment procedure. (I) Survival curve of the mice treated with RT/RT + UK5099. (J) HE staining of tissue slices from mice treated with RT/RT + UK5099. The asterisk in the figure presents the necrosis zones. (K) IHC analysis of the mouse tumors. The data are presented as the mean ± SEM. *P < 0.05, **P < 0.01, compared with the control group; UK1: 10 μm UK5099; UK2: 100 μm UK5099; Vi: control virus group; MPC: MPC1/2 overexpression; UK5099: mice treated with UK5099 (6 mg/kg BW); RT: radiotherapy. All experiments were performed more than 3 times. The bars in the HE staining image are 1 mm, and those in the IHC image are 50 μm. All mice were burdened with tumors in the right shoulder area, which is marked by the red circle.

^18^F-FDG-PET-CT and RT are widely used in clinical settings and are dependent on glycolysis. The phenotypes of PCa and benign prostate cell proliferation were differentially induced by low-dose transient MPC blockade. We used an MPC inhibitor to investigate the changes in ^18^F-FDG-PET-CT and RT in PCa. Subcutaneous tumors were detectable by ^18^F-FDG-PET-CT after UK5099 injection, whereas tumors in the control group were not detectable with ^18^F-FDG-PET-CT. Remarkably, after observation at different time points from 15 min to 150 min, the most significant change was apparent after 1.5 h of UK5099 treatment (**Figure 4A**); therefore, the tumor was easier to detect than the surrounding benign tissue (**Figure 4A and 4B**). The maximum and standard uptake values (SUVmax and SUVavg, respectively) were both elevated in tumor tissues after UK5099 treatment (**Figure 4C**). These results were confirmed by the finding that PCa was more readily traced after UK5099 injection (**Figure 4D**).

Moreover, UK1 caused RT sensitization of PCa cells, whereas in BPH-1 cells, the viability decreased to a lesser extent than that in DU145 and C4-2B cells (**Figure 4E and Supplementary Figure S12**). Moreover, DU145 cells, which are more aggressive and malignant, were more sensitive to RT than C4-2B cells (**Figure 4E and Supplementary Figure S6**). *In vivo* studies showed similar results: UK5099 [6 mg/kg body weight (BW)] + RT treatment decreased the tumor size the most in the subcutaneous tumor group (**Figure 4F and 4G**), whereas no significant BW decrease was observed in the UK5099 + RT treatment group, as compared with the group treated only with RT (**Figure 4H**). In agreement with this finding, the survival times of the mice treated with UK5099 + RT were significantly longer than those of the mice treated only with RT (**Figure 4I**). These results were further confirmed by HE and IHC staining, which indicated significantly augmented necrosis after UK5099 + RT treatment (**Figure 4J**, *P* < 0.05).

These results indicated that the different reactions induced by MPC blockade sensitized PCa to detection and sensitized refractory PCa to RT, thus potentially providing novel targets and a theoretical foundation for the clinical diagnosis and treatment of PCa.

### VDAC1-MPC interaction in PCa

**Figure 5:**
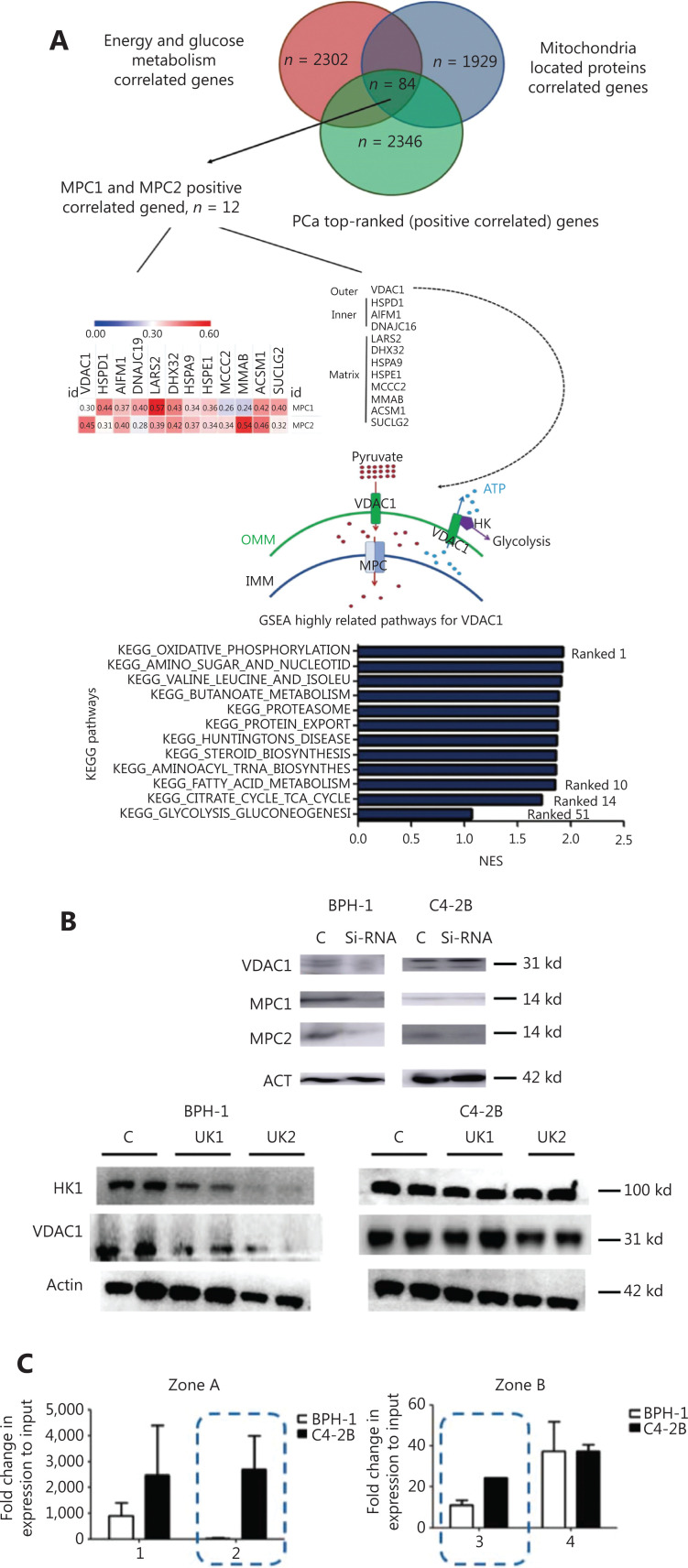

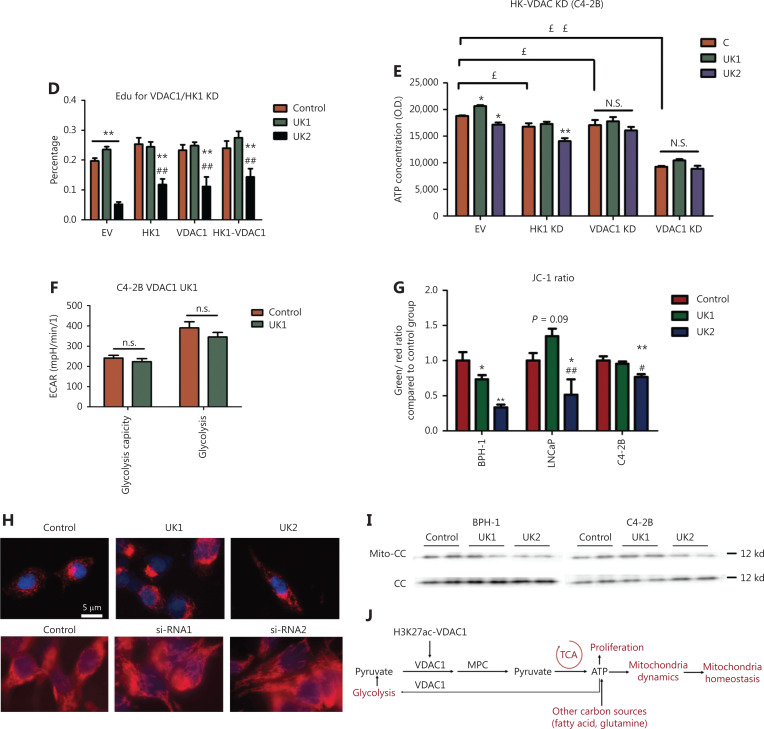
VDAC1-MPC-mitochondrial glycolysis regulation. (A) Mitochondrial genes associated with MPC1 and MPC2 expression in PCa. (B) VDAC1 exhibited differential changes with transient blockade of mitochondrial pyruvate influx in BPH-1 and C4-2B cells. (C) ChIP-qPCR analysis of H3K27ac epigenetic regulation of the VDAC1 promoter zone in benign prostate and PCa cells, performed as described in **Supplementary Figure S4C**. (D) Proliferation changes in response to UK5099 treatment of VDAC1 and HK1 KD cell lines. (E) Effects of VDAC1 and HK1 downregulation on the promotion of ATP. (F) The promotive effect of UK1 on ECAR is eliminated by VDAC1 KD. (G) Changes in mitochondrial membrane potential. (H) Changes in mitochondrial dynamics after treatment with MPC inhibitor or MPC2 siRNA. (I) Effects of the MPC inhibitor on the release of cytochrome c in BPH-1 and C4-2B cells. (J) Schematic of VDAC1-MPC-mitochondria-glycolysis regulation. The data are presented as the mean ± SEM. *P < 0.05, **P < 0.01, compared with the control group; ^#^*P* < 0.05, ^##^*P* < 0.01, compared with the UK1 group; ^£^*P* < 0.05, ^££^*P* < 0.01, compared with each group; UK1: 10 μm UK5099; UK2: 100 μm UK5099; EV: empty vector; MPC: MPC1/2 overexpression. All experiments were performed more than 3 times. The bars are 50 μm.

To determine the potential mechanism through which benign prostate cells and PCa cells react differently to transient MPC blockade, we selected MPC-associated genes for a gene screening study (**Figure 5A**). Specifically, VDAC1 was identified as the most closely related gene or channel associated with the MPC complex. VDAC1 is located on the mitochondrial outer membrane and acts in concert with MPC in pyruvate transport. Moreover, GSEA showed a close relationship between VDAC1 and energy metabolism, thus suggesting that it plays a role in glycolysis and the TCA cycle (**Figure 5A**). After detection of key enzymes involved in glycolysis with Western blot (**Figure 5B and Supplementary Figure S13**), HK1 (but not HK2) and VDAC1 were found to be expressed differently in BPH-1 and C4-2B cells after transient mitochondrial pyruvate blockade by UK5099 treatment as well as MPC2 siRNA treatment.

Assuming that the expression differences were caused by epigenetic modifications, we analyzed the public database Washu Epigenome Browser. As expected, VDAC1 in normal prostate cells exhibited a single sharp peak near the promoter zone, colocalizing with the signal from H3K27ac (a mark of activated promoters). However, in the PCa cell lines LNCaP and C4-2B, we observed 2 additional sharp peaks near the promoter zone, which might have represented an enhancer of the promoter of VDAC1 expression (**Supplementary Figure S13**). Consistently, chromatin immunoprecipitation (ChIP)-qPCR showed a significant increase in VDAC1-H3K27ac in C4-2B cells (**Figure 5C**).

HK1 is a VDAC1 conjugate with important roles in the metabolic connection between the TCA cycle and glycolysis. VDAC1-HK1 complex is crucial to metabolic balance^25^. After the knockdown of VDAC1 and HK1, the pro-proliferative effects were diminished (**Figure 5D**), and the ECAR and ATP levels were also altered (**Figure 5E and 5F**). Therefore, the VDAC1-MPC interaction plays a major role in the different reactions in BPH-1 and C4-2B cells.

### MPC inhibition alters mitochondrial homeostasis

VDAC1 is considered a gatekeeper for mitochondrial homeostasis. The GSVA results for mitochondrial mRNA showed substantial elevated in PCa cells (**Figure 1B**). In addition, VDAC1 plays a critical role in mitochondrial homeostasis. The MMP is regulated by the cellular ATP level^26,27^. MPC blockage tended to decrease the MMP level in BPH-1 cells and in UK2-treated C4-2B cells (**Figure 5G and Supplementary Figure S13**). After knockdown of the expression of VDAC1, thus blocking the VDAC1-MPC interaction, the C4-2B cell line showed a significantly lower MMP. This MMP change might have been caused by the decreased ATP concentration, and it subsequently contributed to the mitochondrial dynamic process (**Figure 5H and 5I**), the most important mitochondrial homeostasis pathway. We then used cellular total cytochrome c as a control to balance the loading protein and to detect the mitochondrial cytochrome c level (**Figure 5I**). In this way, we were able to observe the mitochondrial apoptosis induced by the decrease in MMP and cytochrome c release.

Together, our results indicated that mitochondrial homeostasis induced by MPC is dependent on the ATP-MMP-mitochondrial fission axis and is important for MPC-induced regulation of cell fate. The VDAC1-MPC-mitochondrial homeostasis-glycolysis regulation pathway contributes to different reactions in PCa and benign prostate cell lines, and plays an important role in the MPC-dependent energy shift (**Figure 5J**).

## Discussion

The main findings of this study are that non-carcinoma prostate cells and PCa cells react differently to the blockade of mitochondria pyruvate influx, and that the transient promotion of glycolysis induced by this blockade increases the sensitivity of PCa cells to ^18^F-FDG-PET-CT and RT *In vivo*. We refer to this phenotype as GP, which is induced by the transient inhibition of mitochondrial pyruvate influx and is profoundly reprogrammed in PCa. In addition, associated changes in the axis of VDAC1-MPC-mitochondrial homeostasis-glycolysis regulation modify this metabolic rewiring.

**Figure 6:**
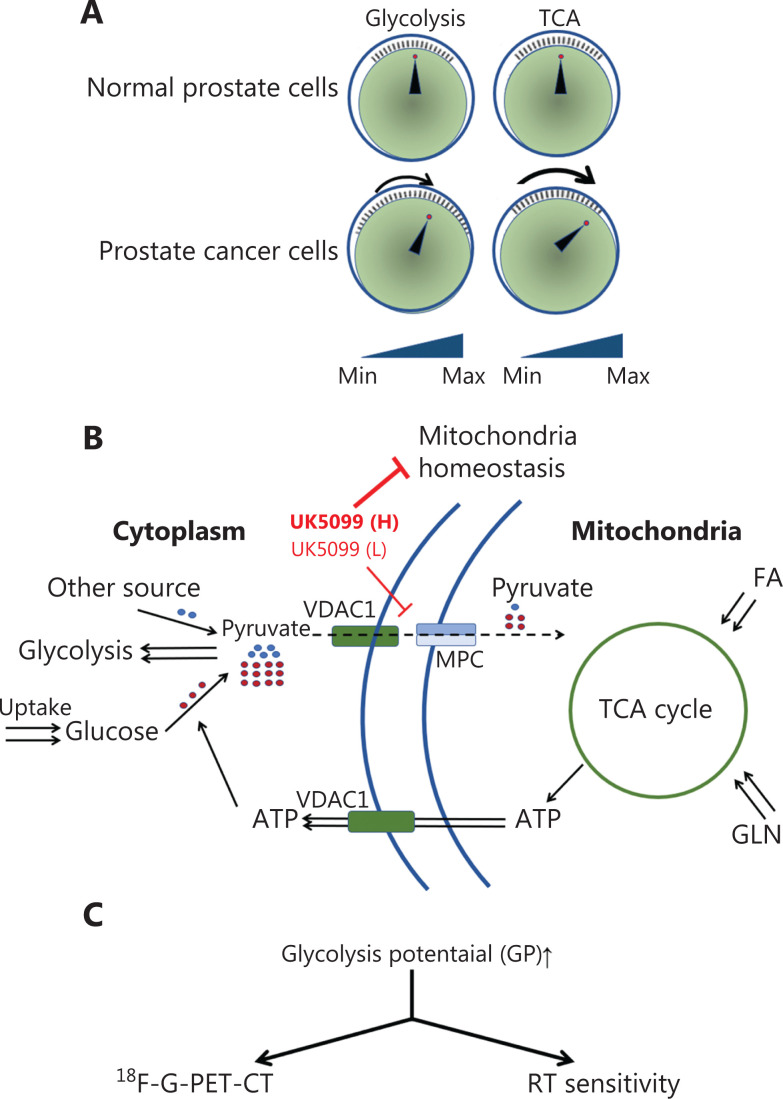
Scheme of glycolytic potential and the relationship between glycolysis and the TCA cycle in PCa cells. (A) Analysis of metabolic activity in this research suggests that despite the elevated TCA level and diminished glycolysis level, the scale of glycolysis is greater in PCa cells than normal cells. (B) Scheme of the role of MPC in cellular glucose metabolism. (C) Clinical practice with GP. GP can be used to increase ^18^F-FDG-PET-CT sensitivity and RT treatment of PCa.

Benign prostate cell lines and malignant prostate cell lines responded differently to the inhibition of pyruvate uptake into mitochondria with low-dose MPC inhibitor as well as MPC2 siRNA, owing to variations in ATP levels. This apparent difference was due to the regulation of VDAC1, an enzyme that is anchored on mitochondria and normally delivers metabolites across the mitochondrial membrane, and is a key enzyme involved in mitochondrial-associated apoptosis^28^. Strikingly, GP might add value to the ‘switch model’ of glycolysis and the TCA cycle in tumor metabolism (**Figure 6A and 6B**). These observations highlight an enhanced connection between glycolysis and the TCA cycle involved in tumor cell adaptation to mitochondrial pyruvate deficiency, thereby providing a new rationale for the use of drugs to increase ^18^F-FDG-PET-CT sensitivity as well as RT sensitivity. This increase in sensitivity might be useful in the diagnosis and treatment of PCa in clinical settings.

Our data revealed that although PCa has been demonstrated not to use glycolysis rather than TCA cycle to produce energy, the connection between glycolysis and the TCA cycle is more prominent in PCa cells than benign prostate cells. However, in early stages of PCa, such gene deficiencies may not be readily observed, these gene deficiencies contributes to the special metabolic phenotype of PCa. As shown in this research, ATP delivery from the TCA cycle for glycolysis was significantly elevated in PCa cells, thus potentially enabling increased use of glycolysis. Although the amount of ATP yielded by oxidative phosphorylation (OXPHOS) is 18 times greater than that generated *via* glycolysis, the speed of ATP production in glycolysis is 100 times that in OXPHOS^4,29^. Moreover, glycolysis is a topic covered in every biochemistry course because of its central role in biological metabolism^30^. This process produces ATP, generates biosynthetic precursors and macromolecules, and tolerates stresses associated with malignancy^5,31^.

Proliferating cells metabolize glucose primarily through glycolysis, excreting large amounts of carbon in the form of ethanol, lactate, acetate, and butyrate^32^. This enhanced connection between glycolysis and the TCA cycle can provide sufficient energy when cancer cells are stimulated by microenvironmental changes, thus allowing cancer cells to tolerate nutrient depletion by catabolizing macromolecules from inside or outside the cell. Under hypoxic conditions, glutamine oxidation has been reported to maintain the TCA cycle and cell survival with MPC deficiency^15^. In another report, researchers have found that MPC re-expression inhibits cell proliferation, invasion, and migration *in vitro* under hypoxic conditions (1% oxygen) without glutamine supplementation^33^. These results together indicate that MPC loss might contribute to tumor development under hypoxic conditions, which is induced by fast growth. Moreover, the transcription factor hypoxia-inducible factor 1 (HIF-1) is negatively correlated with MPC1 expression^34,35^. Thus, glycolytic potential might also be an important protective characteristic for carcinoma cell growth under hypoxic conditions.

The concept of GP might supplement the switch model of the TCA cycle and glycolysis presented in 2016^5^. According to a commonly held viewpoint, cancer cells undergo a switch from glucose OXPHOS to glycolysis. However, an increasing number of researchers now argue against such a switch. As shown, tumors appear to simultaneously enhance both glycolysis and glucose oxidation relative to that in the surrounding tissue^5^, whereas the increase in glycolysis is greater than that in OXPHOS. However, these findings still cannot fully explain the metabolic rewiring that occurs in cancer cells, in which glycolysis does not play a predominant role in energy production. According to our GP model, rather than the previous model, the influence of the glycolysis switch might be greater in cancer cells than in normal prostate cells, even if the tumor does not favor glycolysis over the TCA cycle to produce energy. Tumors might induce different types of metabolic rewiring according to their surroundings.

Our study provides additional insight into the clinical utilization of the different reactions induced by UK5099 as well as MPC2 siRNA. ^18^F-FDG-PET/CT scans are a valuable adjunct for characterizing cancer in clinical work, particularly in clinical screening^36,37^. However, in non-glycolytic tumors, diminished glucose uptake contributes to the lower sensitivity of PET signals from 2-deoxy-2-[^18^F]-FDG tracers, particularly in localized tumors. In PCa, elevated glucose uptake is observed only in advanced tumors^38,39^. Several studies have demonstrated the utility of PET as a promising tool for predicting patient survival in PCa^37^. As shown in our results, low-dose inhibition of the MPC indeed increased glucose uptake *in vitro*. Our study also reports the first evidence that MPC inhibition results in activation of the Warburg effect and enhances [^18^F] FDG signals in PCa mouse models. As expected, UK5099 decreased the tumor size in the subcutaneous model (**Figure 4G**). Moreover, because the GP differed between normal cells and carcinoma cells, the inhibition of the MPC tended to sensitize PCa cells to RT in both *in vitro* and *in vivo* studies (**Figure 4E–4G and Supplementary Figure S12**). This finding might have been caused by the increased glycolysis induced by MPC inhibition. Thus, GP stimulation might be a good choice for patients with PET-CT-insensitive non-glycolytic cancers.

Strikingly, our study revealed that VDAC1-MPC-mitochondrial homeostasis-glycolysis regulation might be the mechanism underlying the different responses between non-carcinoma and PCa cells. VDAC1 allows for pyruvate transport from the cytoplasm into the mitochondrial outer membrane. Although the MPC is partly blocked, some pyruvate can still cross the inner membrane for ATP production and be delivered outside the mitochondria by VDAC1. Moreover, as previously reported, MPC downregulation enhances energy production from fatty acids or glutamine, and oxidation also occurs in mitochondria^15,24,40,41^. Mitochondrial dynamics is the most critical pathway of mitochondrial homeostasis and is regulated by ATP levels and the MMP^42^. As demonstrated by a recent study in yeast, pyruvate metabolism depends on both VDAC1 and MPC, which regulate whole-cell cellular processes and biochemical interactions^43^. When MPC is mostly blocked or VDAC1 cannot be increased to a proper level, glucose-induced mitochondrial homeostasis is interrupted, thus leading to cell death and downregulation of proliferation.

VDAC1 is a crucial influence on cell fate, which is particularly relevant to cancer cells^44^: it controls the metabolic crosstalk between mitochondria and cellular plasma^25^. Thus, it links the TCA cycle and glycolysis by transporting ATP and other metabolites^45^. As observed, VDAC1 can associate with various enzymes, including HK and apoptotic proteins. By the direct coupling of ATP from mitochondria to glucose uptake *via* VDAC1-bound HK, mitochondria regulate glycolytic flux *via* the TCA cycle and ATP synthase, thereby delivering adequate energy and metabolite precursors to tumor cells to meet biochemical requirements. Thus, the glycolytic pathway is regulated *via* energy coupling resulting from the formation of the VDAC1-bound HK complex^46,47^. In our study, when pyruvate influx into mitochondria was transiently blocked by the inhibitor or MPC2 siRNA, HK1 and VDAC1 decreased significantly in the BPH-1 cell line but not in the C4-2B cell line. In this way, the HK1-VDAC1 complex, which binds more strongly in PCa cells, tends to stabilize the MPC complex and, in a feedback response, normalizes mitochondrial homeostasis to support proliferation.

In this study, when mitochondrial pyruvate influx was blocked by low-dose UK5099, cancer cells tended to use more glucose to produce pyruvate, which can be used in the TCA cycle to produce ATP and maintain mitochondrial homeostasis. Thus, although the total TCA metabolites were decreased in both BPH-1 cells and C4-2B cells, the TCA cycle metabolites from glucose were increased in C4-2B cells but not BPH-1 cells (the schematic is shown in **Figure 6B**). Compared with noncancer cells, PCa cells presented a high level of VDAC1-H3K27ac modification in the enhancer zone (**Figure 5C**). Thus, VDAC1 transcriptional activity increased in cancer cells and responded more quickly to nutrient starvation. Our findings indicate the utility of UK5099 or other MPC inhibitors in future clinical work. However, the detailed mechanism requires additional study.

VDAC1 plays important roles in mitochondrial homeostasis, a process critical for determining the cellular fate. The decrease in pyruvate influx into mitochondria blocks mitochondrial fusion, downregulates the MMP, and decreases cellular ATP levels^48^. Cytochrome c is released from the inner membrane into the cytoplasm during blockage^48–51^. However, overexpression of MPC did not reverse this effect. This inhibition of the proliferative effect by MPC overexpression has been reported in colon cancer^16^. However, no agonist is available for MPC; therefore, further study is necessary. The specific effect of the MPC on the development of cancer remains poorly understood, although some studies have shown that it is closely associated with the stemness of cancer cells^16^.

## Conclusions

Blockade of pyruvate influx into mitochondria increases glycolysis in PCa and thus sensitizes PCa to detection and radiotherapy, whereas transient blockade decreases glycolysis levels in benign prostate cells. GP is a novel mechanism underlying PCa progression, and the change in mitochondrial pyruvate influx is a critical target for PCa diagnosis and treatment.

## Data Availability

All data included in this research are available on request. Correspondence and requests for materials should be addressed to F.W. (wangbofengye@163.com).
