# Supplementary material for: Glycolytic potential enhanced by blockade of pyruvate influx into mitochondria sensitizes prostate cancer to detection and radiotherapy
Source: Cancer Biol Med. 2022 Aug 17;19(9):1315–33. doi: 10.20892/j.issn.2095-3941.2021.0638 (PMC9500223; doi:10.20892/j.issn.2095-3941.2021.0638)
Supplement: Supplementary file 1 [file cbm-19-1315-s001.pdf]

Supplementary materials

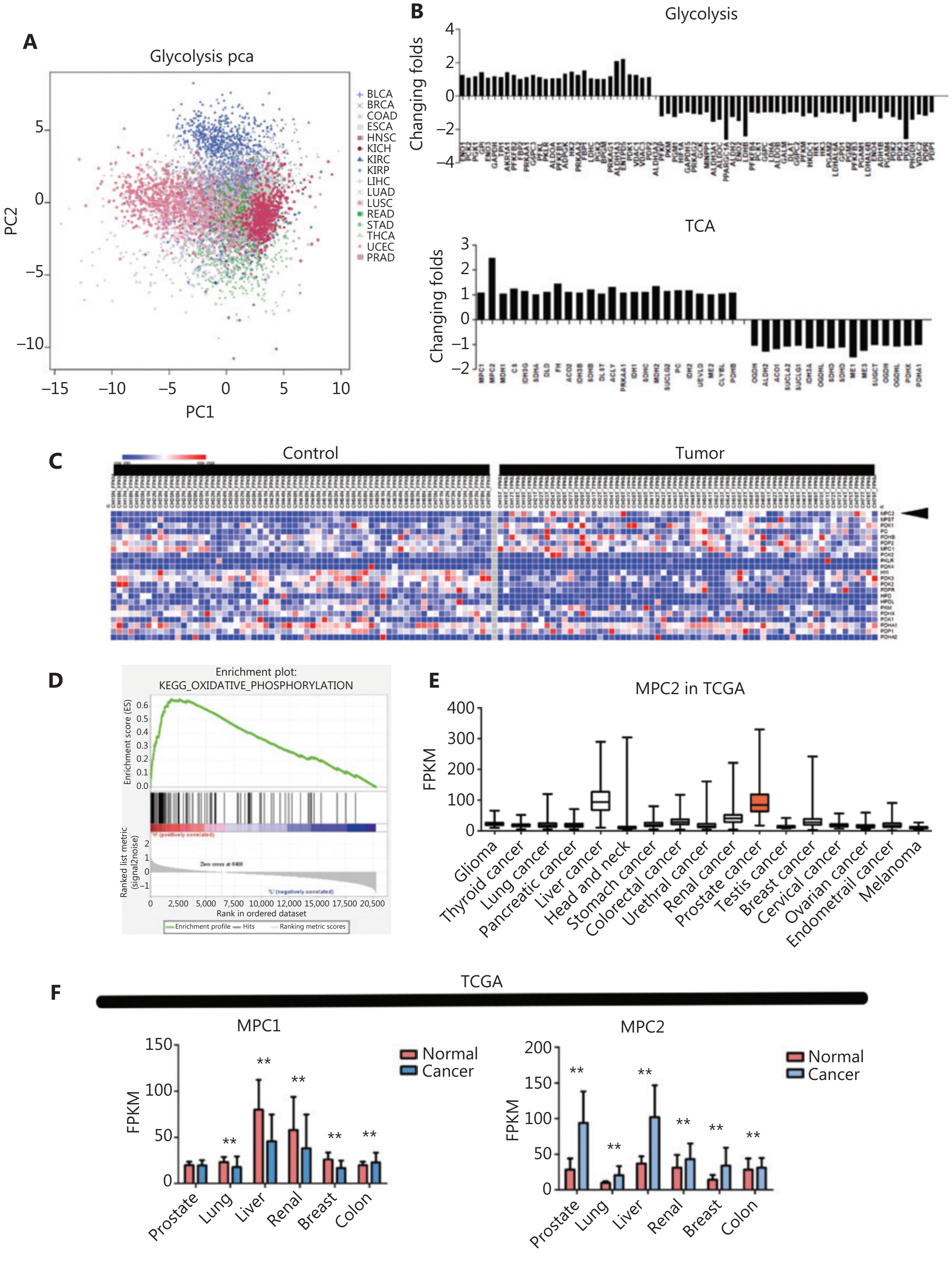

Figure S1 Related to **Figure 1**. Metabolic changes in prostate cancer and MPC expression. (A) Changes in glycolysis and TCA cycle associated mRNA in prostate cancer (relative to **Figure 1A**). (B) Different expression of glycolysis and TCA cycle associated mRNA levels. The fold change

is shown by the ratio of PCa to benign tissue. (C) RNA-Seq data for pyruvate metabolism associated genes in prostate cancer (the black arrow indicates MPC2) and MPC2 expression in different tissues. (D) GSEA of MPC2 mRNA levels and OXPHOS. (E) MPC2 levels in different types of cancers, according to TCGA data. (F) MPC1 and 2 levels in tumor and non-tumor tissues from different types of cancers, according TCGA data. The data are represented as mean ± SEM. ***P* < 0.01, compared with the control group. The data from A-D are from group A.

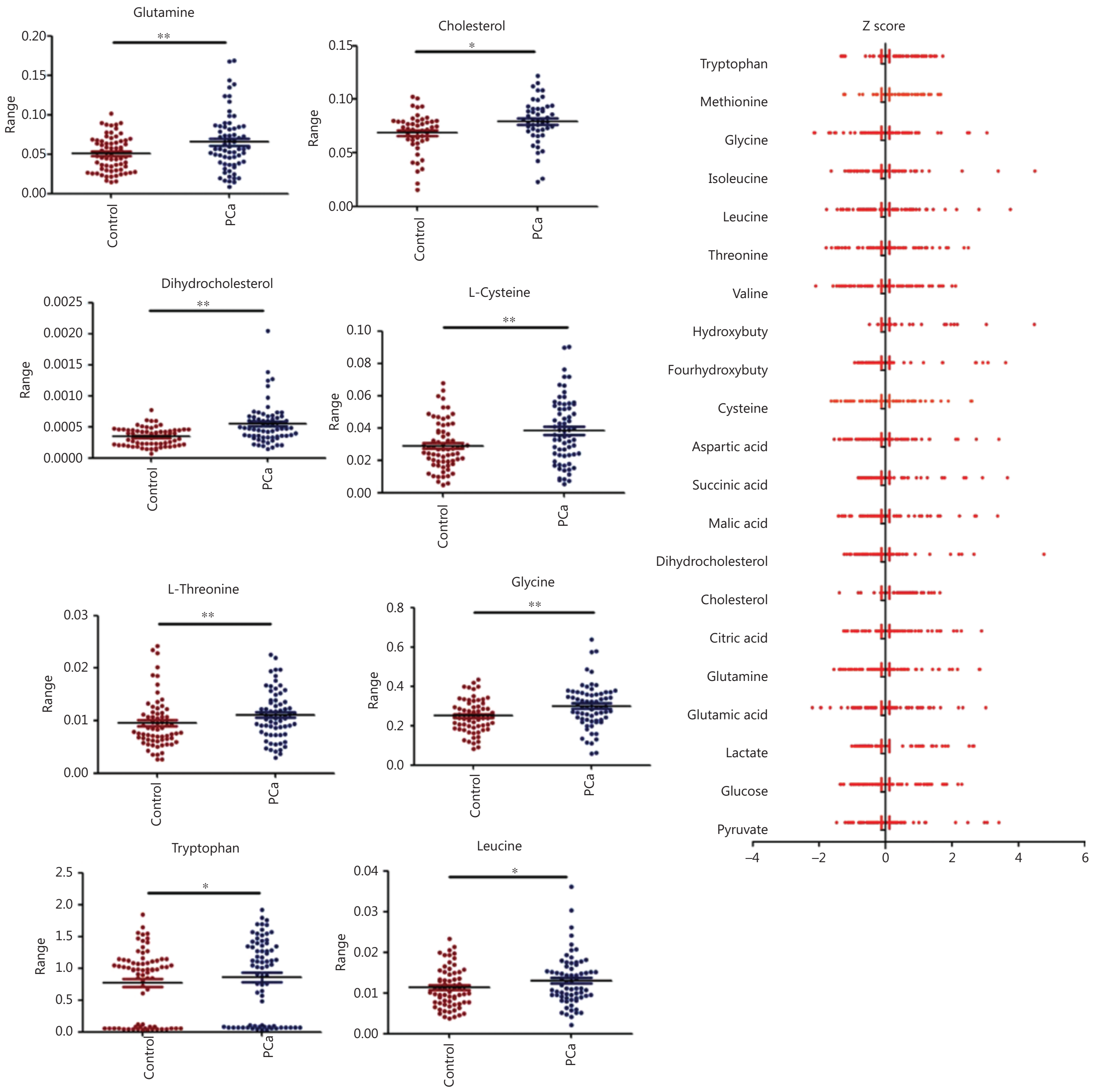

Figure S2 Related to **Figure 1**. Glucose associated metabolic profiling data of prostate cancer and benign prostate tissue. The data are represented as mean ± SEM. **P* < 0.05; ***P* < 0.01, compared with the control group.

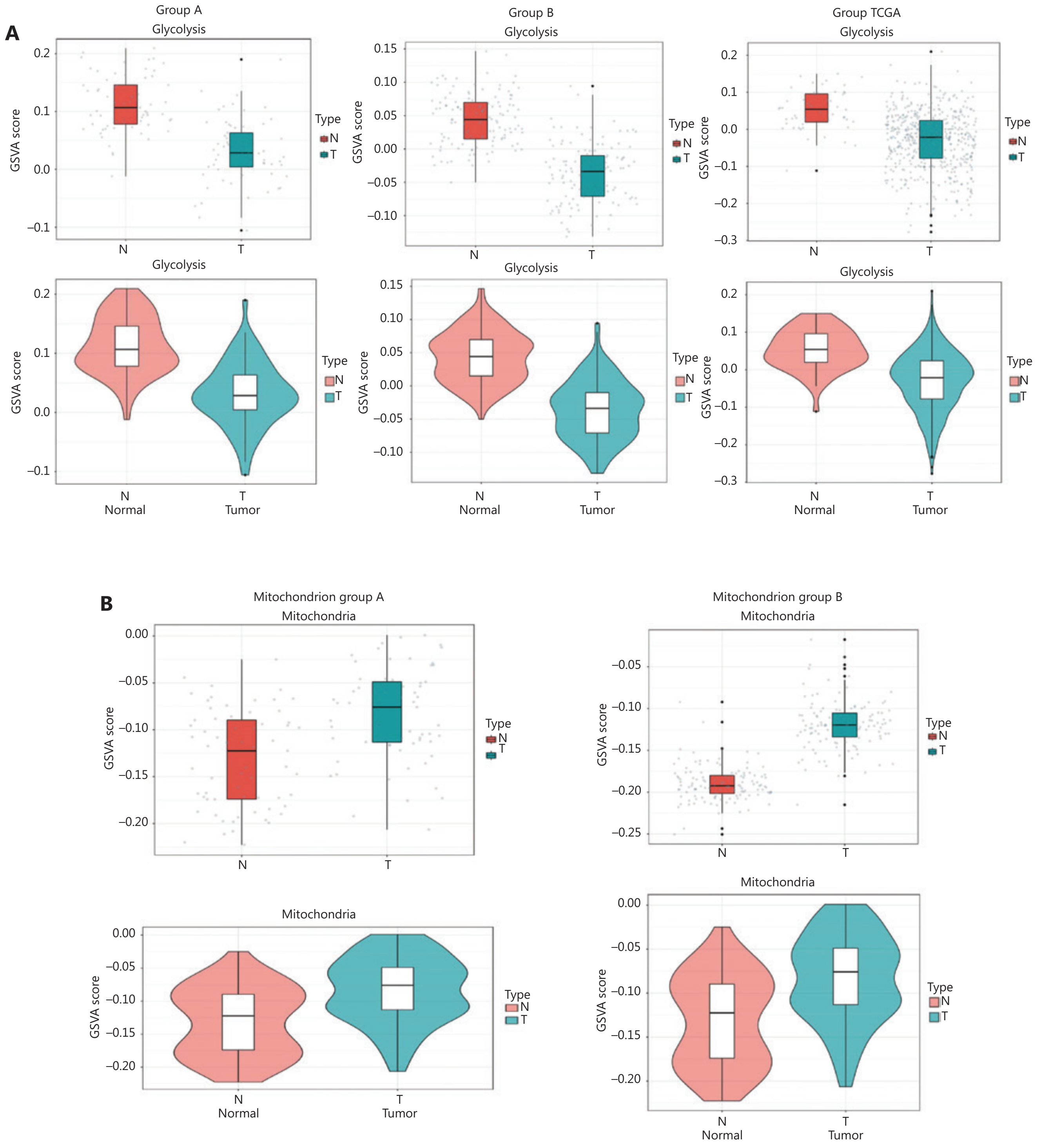

Figure S3 Related to **Figure 1**. (A) Different expression of glycolysis associated mRNA in different groups of patients. (B) Different expression of mitochondrially associated mRNA in different groups of patients.

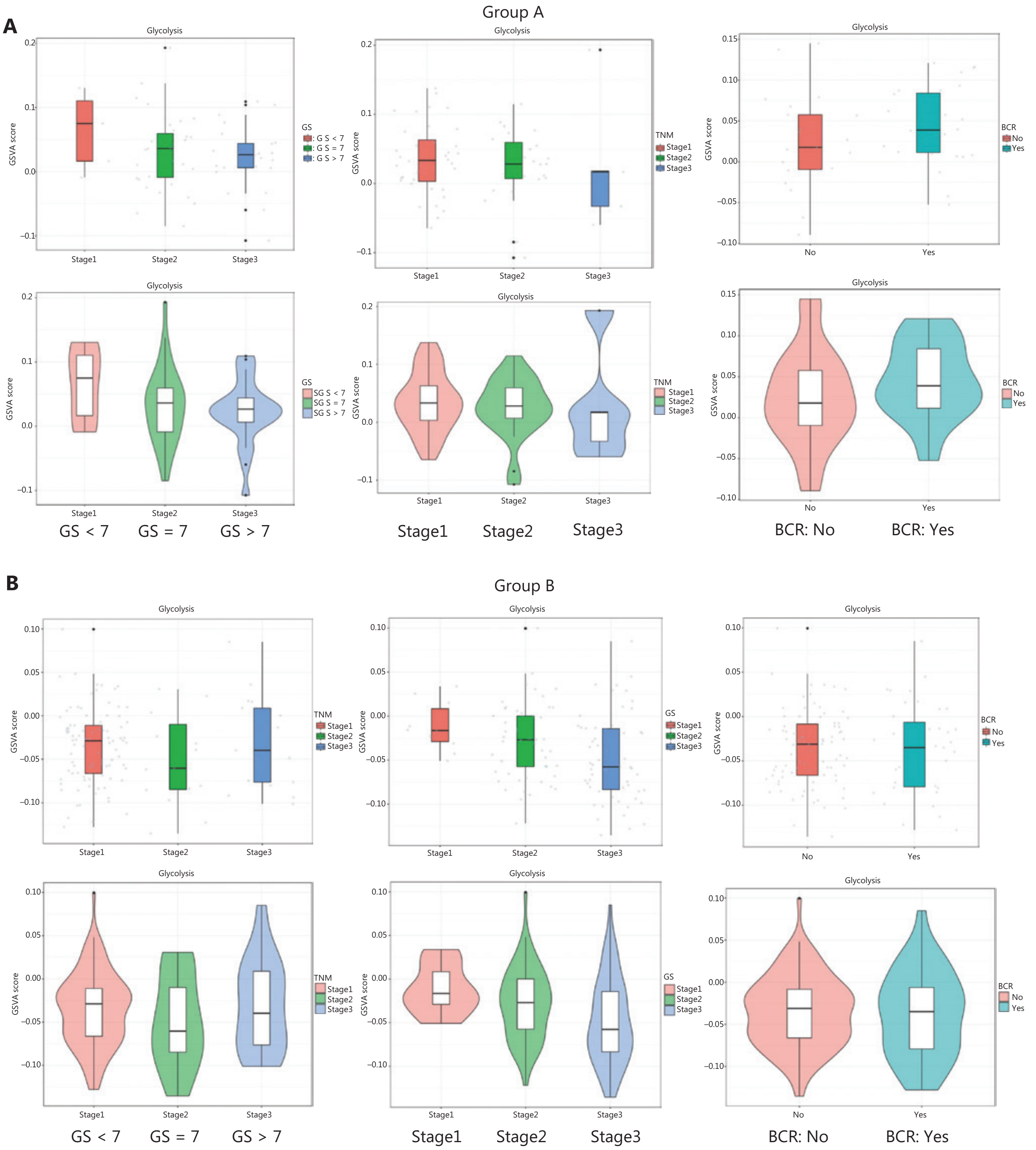

Figure S4 Related to **Figure 1**. Different expression of glycolysis associated mRNA in different groups of patients according to the clinical prognosis, including TNM stages, Gleason score (GS), and BCR. In the GS, TNM stage 1 = T1-2N0M0, stage 2 = T3-4N0M0, stage 3 = TxN1M0.

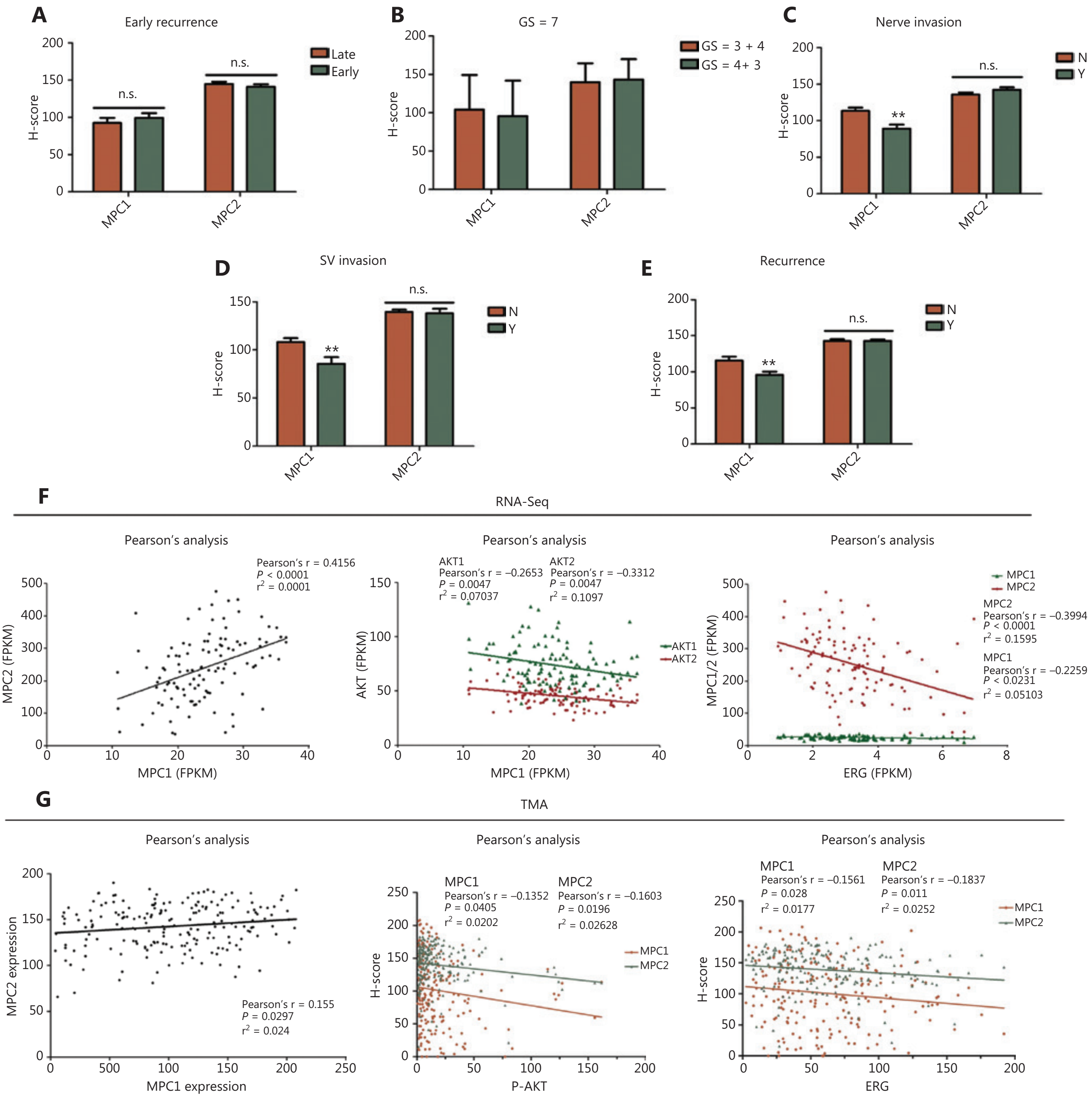

Figure S5 Related to **Figure 1**. (A–E) Different expression of MPC according to different clinical characteristics in group A patients. (F, G) Co-expression between MPC1/2 and the oncogenes AKT and ERG in PCa. The data are from the RNA-Seq data (group B, *n* = 125) or TMA data (*n* = 210) as indicated. The data are represented as the mean ± SEM. ***P* < 0.01.

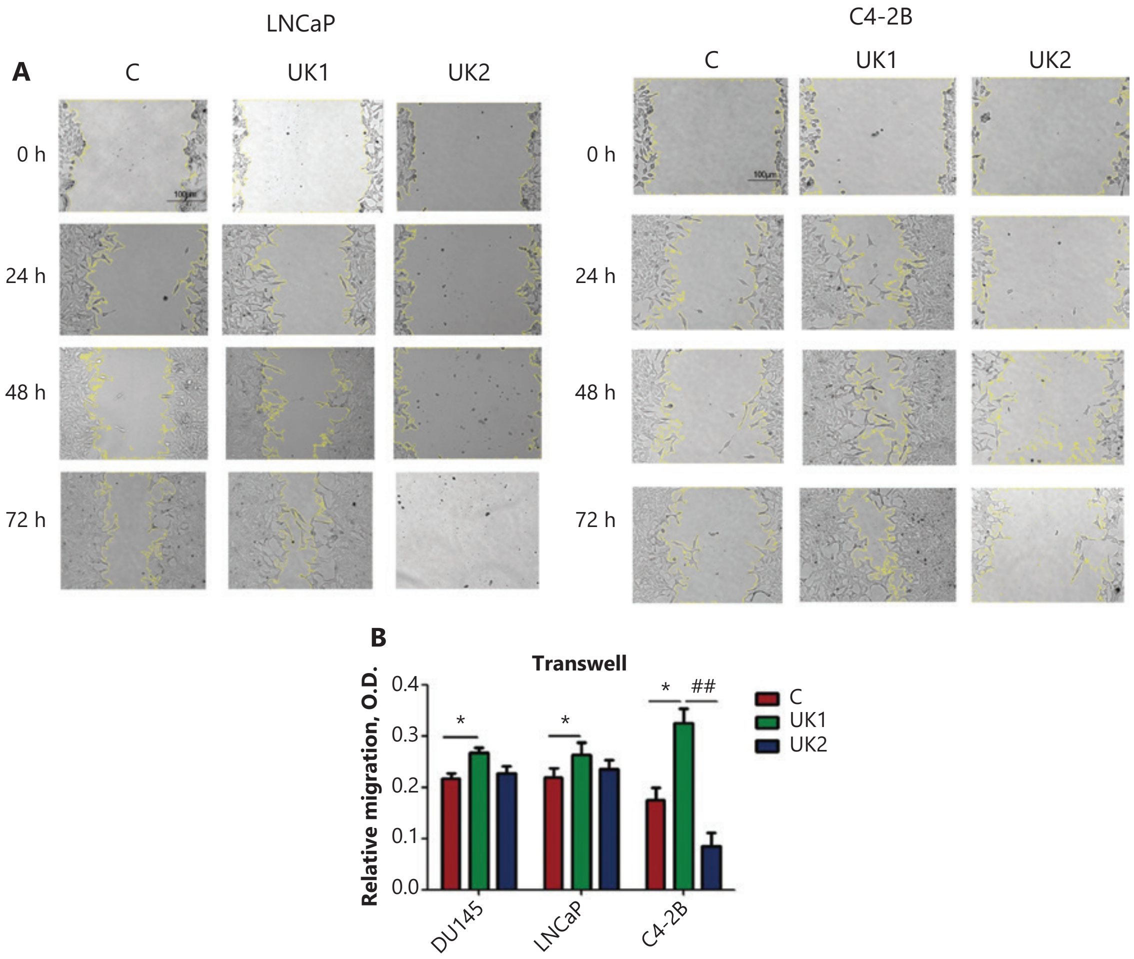

Figure S6 Related to **Figure 2**. Different effects of transient blockage of mitochondrial pyruvate influx in benign prostate cells and prostate cancer cells. (A) Wound healing analysis of LNCaP and C4-2B cell lines with C, UK1, and UK2 treatment. (B) Transwell study of DU145, LNCaP, and C4-2B cell lines with C, UK1, and UK2 treatment. *P < 0.05, ##P < 0.01.

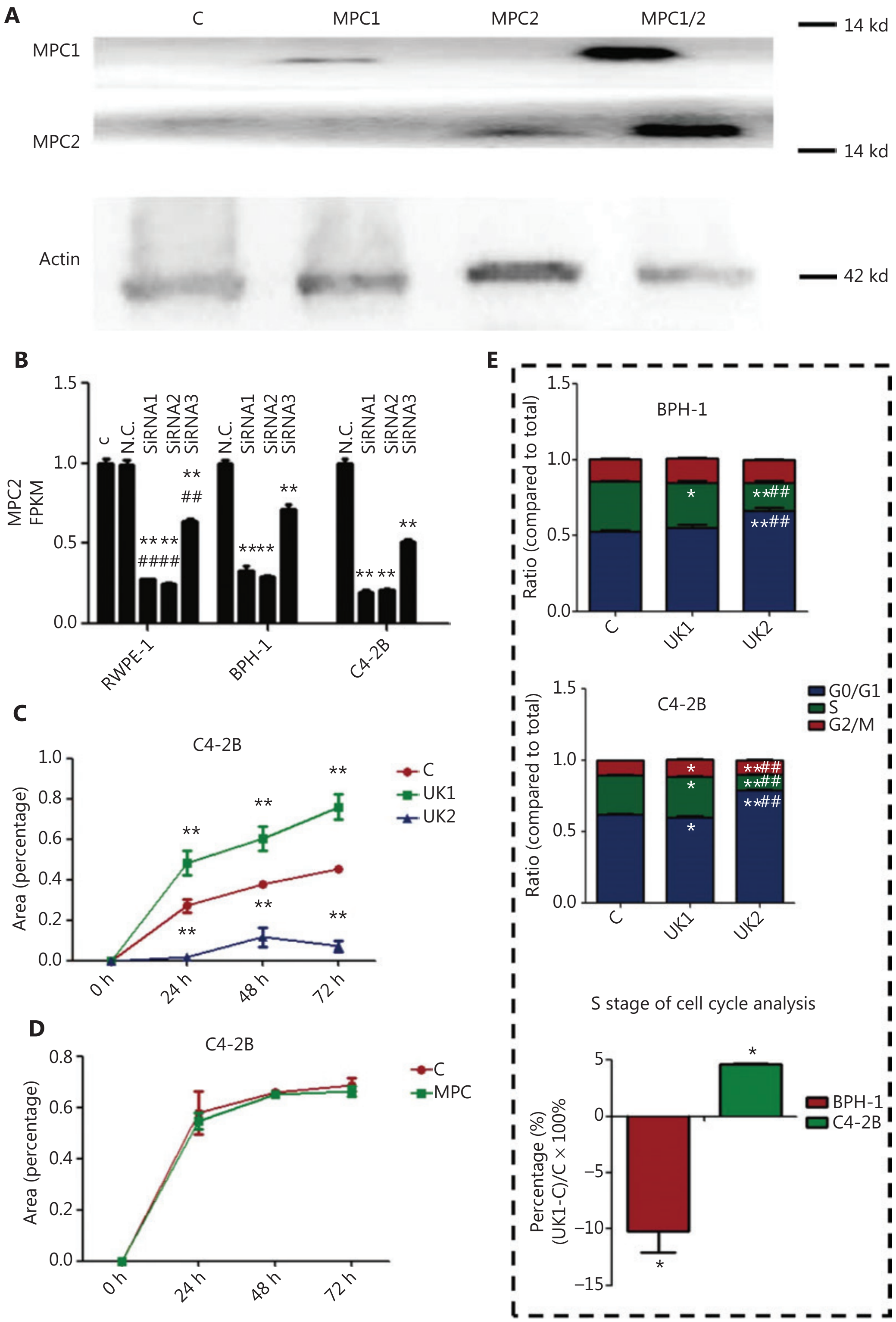

Figure S7 Related to **Figure 2**. (A, B) MPC expression after siRNA treatment. (C) Growth curve of UK5099 treated C4-2B cells. (D) Growth curve of MPC overexpressing C4-2B cells. MPC: MPC overexpression. (E) Cell cycle analysis of different cell lines after treatment with UK5099. The data are represented as mean ± SEM. **P* < 0.05, ***P* < 0.01, compared with the control group, $^{##}P < 0.01$, compared with the UK1 group, UK1: 10 μm UK5099, UK2: 100 μm UK5099, EV: empty vector, MPC: MPC1/2 overexpression. All experiments were performed in more than 3 replicates. The bars are 10 μm.

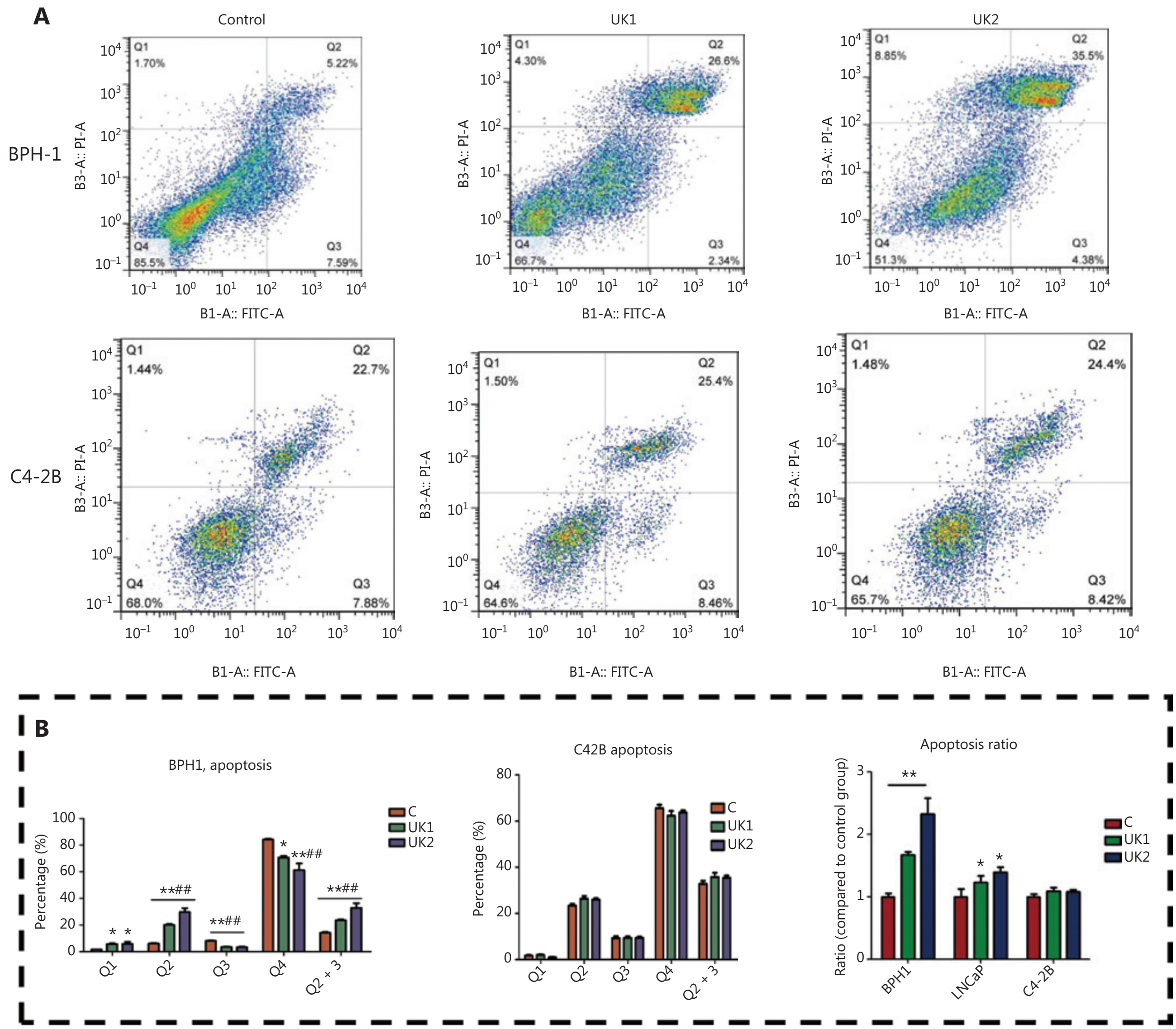

Figure S8 Related to **Figure 2**. Apoptosis, measured by flow cytometry analysis of control, UK1, and UK2 treated cell lines. **P* < 0.05, ***P* < 0.01, compared with the control group, ##*P* < 0.01, compared with the UK1 group, UK1: 10 μm UK5099, UK2: 100 μm UK5099, EV: empty vector, MPC: MPC1/2 overexpression. All experiments were performed in more than 3 replicates. The bars are 10 μm.

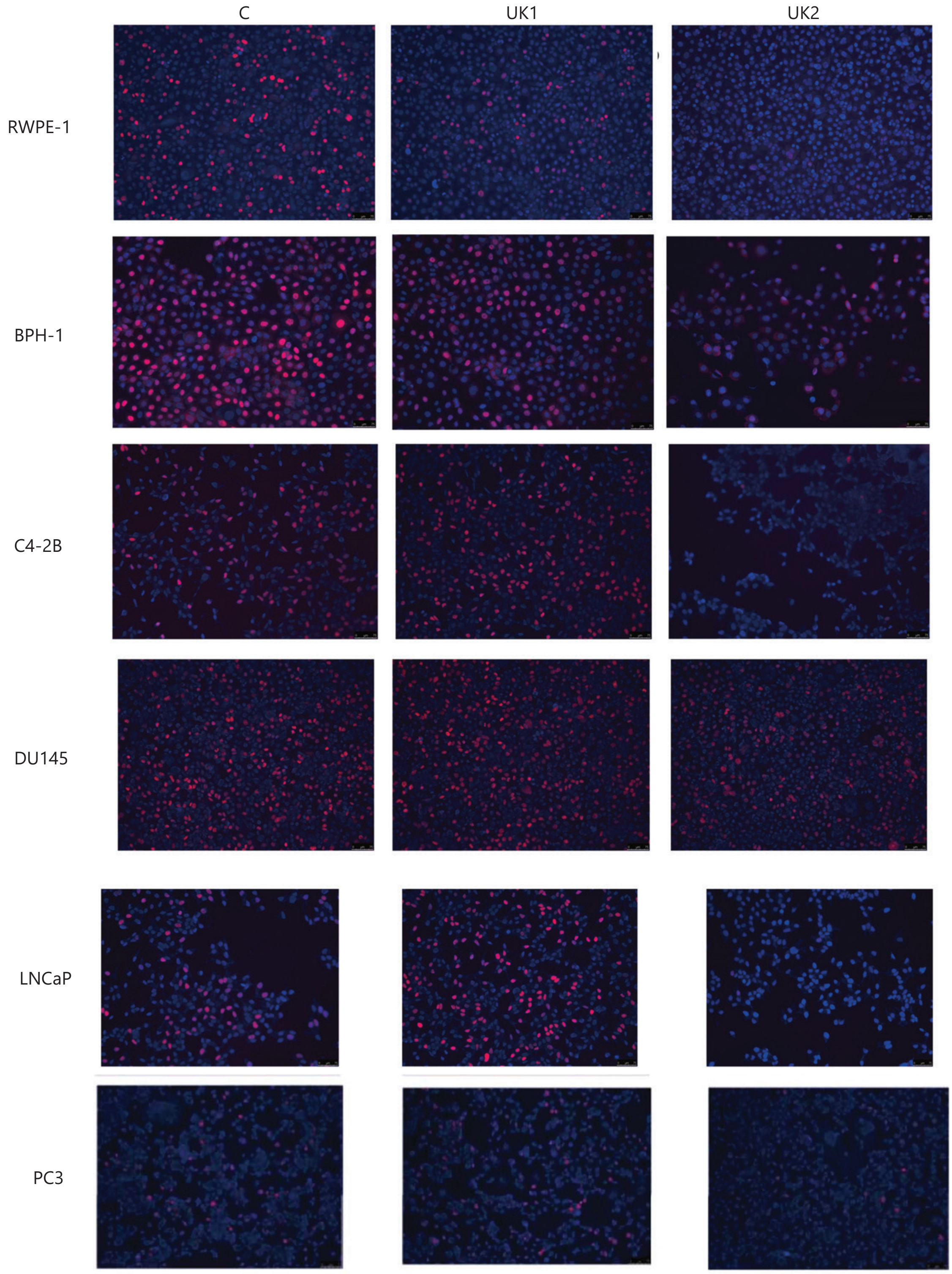

Figure S9 Related to **Figure 2**. EdU measurement in control, UK1, and UK2 treated cell lines.

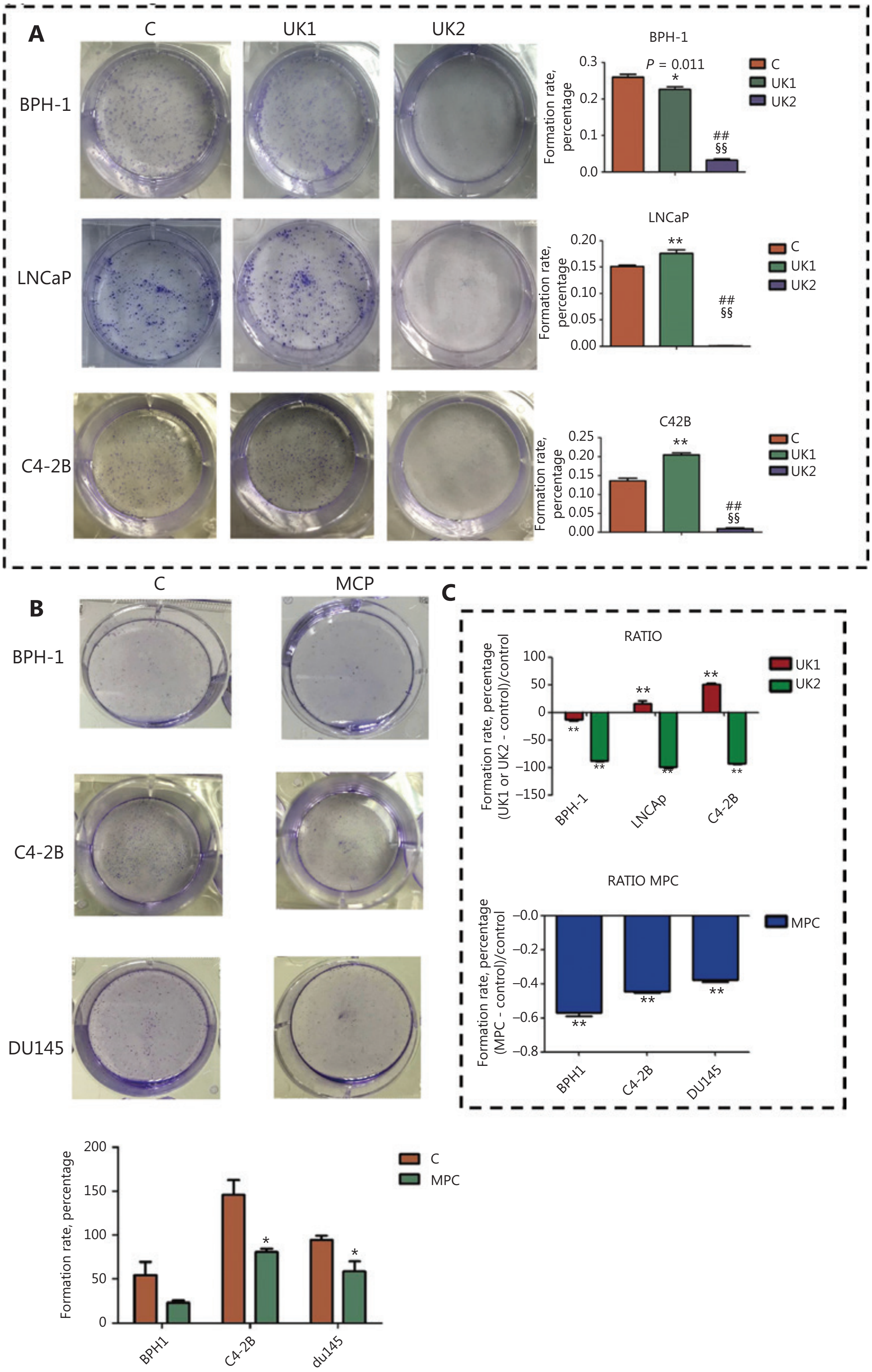

Figure S10 Related to **Figure 2**. Clone formation study. (A) Clone formation study of UK5099 treated different cell lines. (B) Clone formation study of MPC overexpression in different cell lines. (C) Analysis of clone formation study above. $^{*}P < 0.05$, $^{**}P < 0.01$, compared with the control group, $^{\#\#}P < 0.01$, compared with the UK1 group, UK1: 10 μm UK5099, UK2: 100 μm UK5099, EV: empty vector, MPC: MPC1/2 overexpression. $^{\S\S}P < 0.01$.

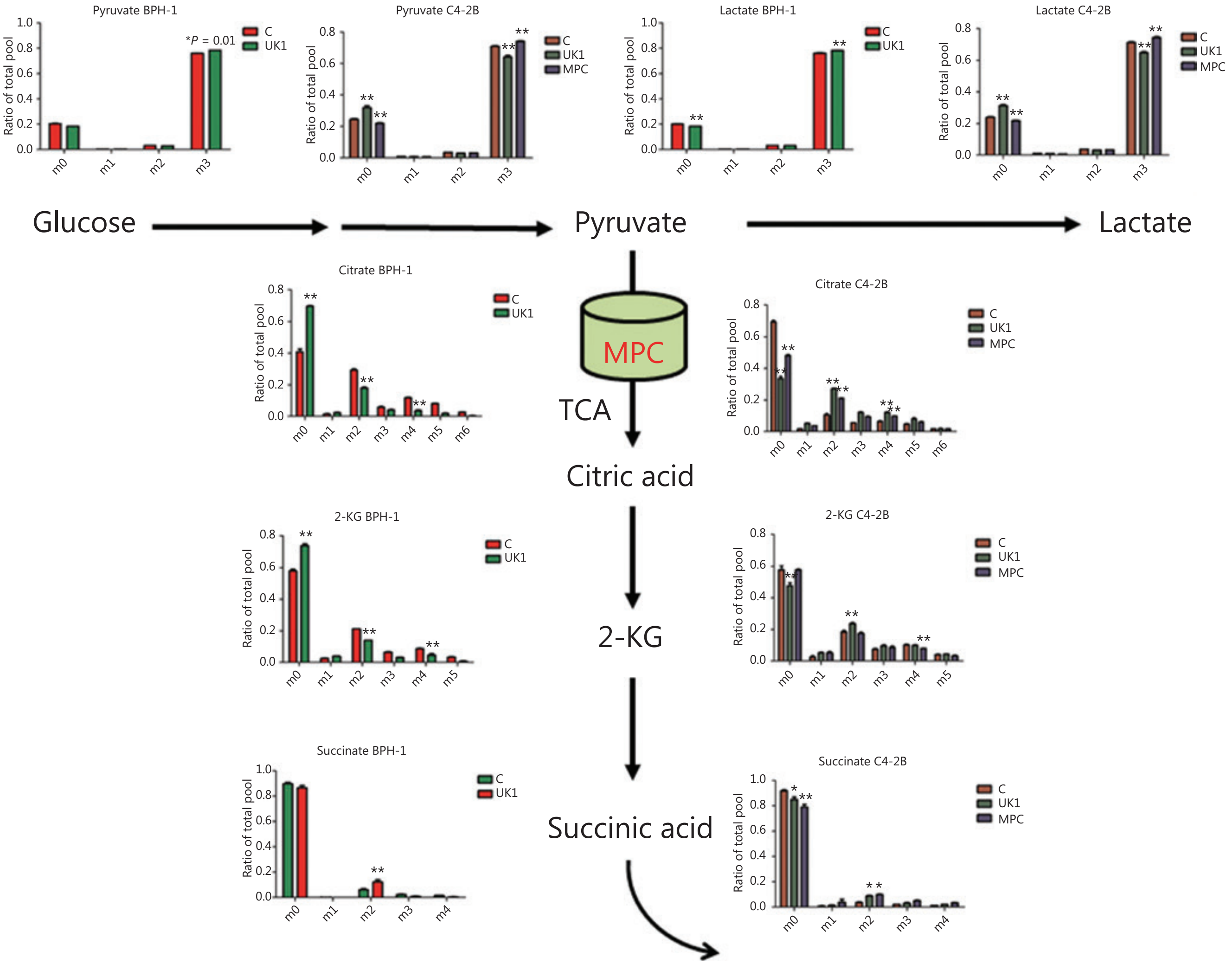

Figure S11 Related to **Figure 3**. D-[U-^{13}C] glucose tracer for the metabolic process in BPH-1 and C4-2B cell lines. *P < 0.05, **P < 0.01, compared with the control group, UK1: 10 μm UK5099, UK2: 100 μm UK5099, EV: empty vector, MPC: MPC1/2 overexpression.

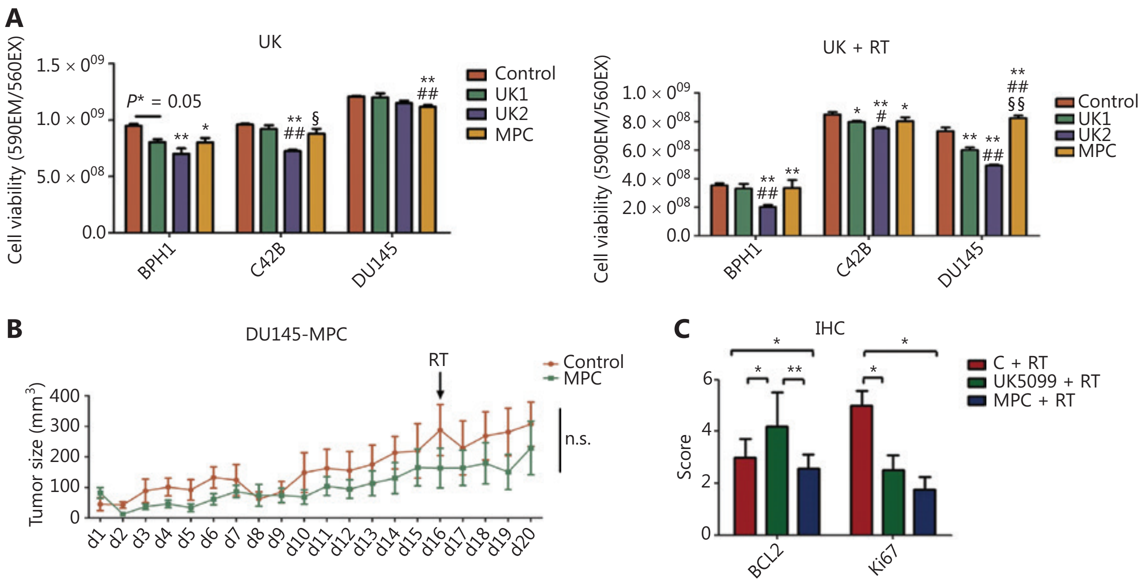

Figure S12 Related to **Figure 4**. *In vivo* study and the application of MPC transient block. (A) UK5099 increases the radiosensitivity of the PCa cells. When treated with UK5099, C4-2B and DU145 present more sensitivity to RT than the BPH-1 cells. (B) The growth curve of a DU145-MPC overexpressing subcutaneous tumor. (C) Analysis of IHC staining of BCL2 and Ki67. The data are represented as mean ± SEM. **P* < 0.05, ***P* < 0.01, compared with the control group, UK5099: mice treated with UK5099 (6 mg/kg BW), MPC: MPC1/2 overexpression. All experiments were performed in more than 3 replicates. #*P* < 0.05, ##*P* < 0.01, compared with the UK1 group; §*P* < 0.05, §§*P* < 0.01.

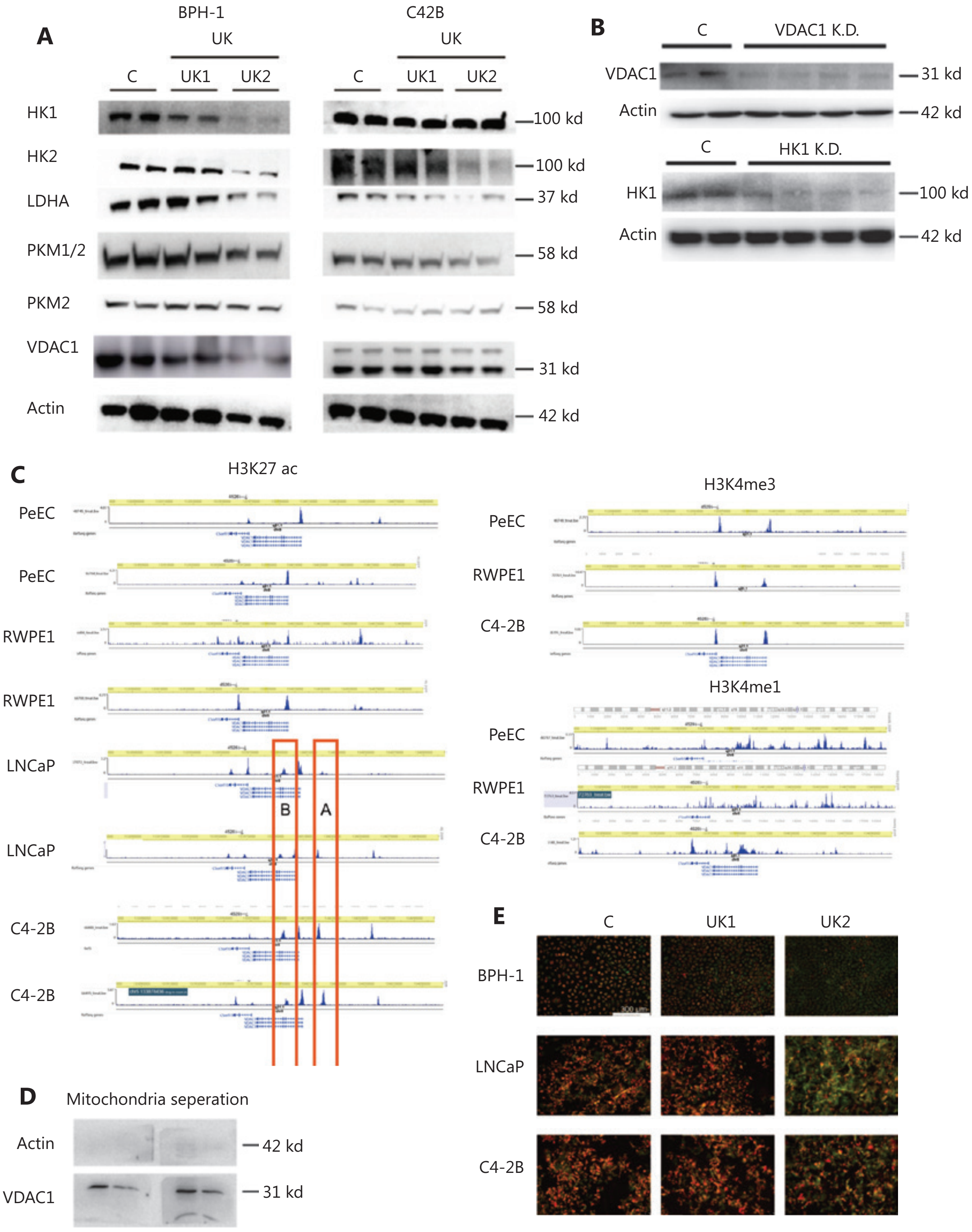

Figure S13 Related to **Figure 5**. Effects of mitochondrial pyruvate influx on mitochondrial homeostasis. (A) Western blot analysis of the screened glycolysis associated key enzymes. (B) Western blot analysis of VDAC1 and HK1 changes after VDAC1 or HK1 downregulation. (C) Epigenetic analysis results from the public database Washu Epigenome Browser. Two high peaks were observed in LNCaP and C4-2B but not in normal prostate cell lines. We designed 2 primers for each zone (A-1, A-2, B-3, and B-4). (D) Western blot analysis of the efficiency of mitochondrial separation. (E) JC-1 fluorescence in BPH-1, C4-2B, and LNCaP cell lines after UK5099 treatment. The data are represented as mean ± SEM. UK1: 10 µm UK5099, UK2: 100 µm UK5099, MPC: MPC1/2 overexpression. All experiments were performed in more than 3 replicates. The bars are 10 µm.

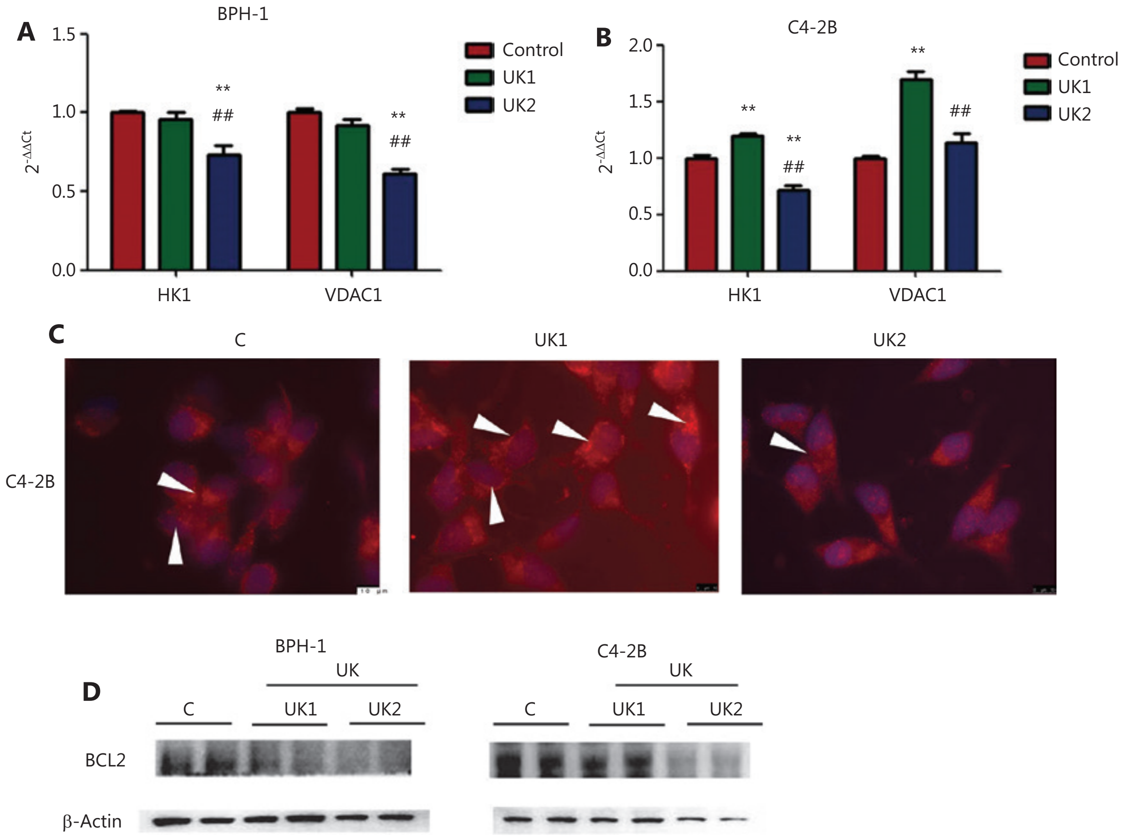

Figure S14 Related to **Figure 5**. (A, B) HK1 and VDAC1 mRNA levels in different cell lines after UK5099 treatment. (C) Cytochrome c (white arrows) changes in C4-2B cell lines observed with immunofluorescence. (D) BCL2 changes in BPH-1 cells and C4-2B cells after low-dose UK5099 treatment. The data are represented as mean ± SEM. $^{**}P < 0.01$, compared with the control group, $^{\#\#}P < 0.01$, compared with the UK1 group, UK1: 10 μm UK5099, UK2: 100 μm UK5099, MPC: MPC1/2 overexpression. All experiments were performed in more than 3 replicates. The bars are 10 μm.

Table S1 Glycolysis associated genes

ALDH3B1
PFKP
PKM
ALDH3A2
ENO1
PGM1
DLD
PCK2
PGK1
GPI
GAPDHS
GCK
MINPP1
ENO3
ALDH3A1
ALDOC
ALDH2
GAPDH
TPI1
ENO2
LDHB
AKR1A1
PCK1
FBP2
ACSS2
G6PC
PDHA1
ALDH3B2
LDHA
ALDOB
ALDH1B1
G6PC3
PFKL
ALDH9A1
PKLR
GALM

Table S1 Continued

ALDOA
DLAT
G6PC2
PFKM
ACSS1
HKDC1
HK1
ADPGK
HK2
HK3
PDHA2
PGAM2
ALDH7A1
FBP1
LDHC
LDHAL6A
PDHB
PGM2
PGK2
PGAM1
LDHAL6B
BPGM
ADH6
ALDH1A3
ADH1A
ADH7
ADH1B
ADH5
ADH4
PGAM4

Table S2 Primers

Primers	
HK1	
Forward primer	GCTCTCCGATGAAACTCTCATAG
Reverse primer	GGACCTTACGAATGTTGGCAA
VDAC1	
Forward primer	ACGTATGCCGATCTTGGCAAA
Reverse primer	TCAGGCCGTACTCAGTCCATC
MPC1 Fwd1	(GTGCGGAAAGCGGCGGACTA)
MPC1 Rev1	(GGCAGCAATGGGAAGACCCCA)
MPC2 Fwd	(TACCACCGGCTCCTCGATAAA)
MPC2 Rev	(TATCAGCCAATCCAGCACACA)
OPA1-1 forward primer	TGTGAGGTCTGCCAGTCTTTA
OPA1-1 reverse primer	TGTCCTTAATTGGGGTCGTTG
AR binding sequence:	
MPC1	
Primer pair 1-1	
Forward primer	GTCCCTATGCACAATGAGTAGC
Reverse primer	ATGGTGCATCCGTTTAGTGGA
Primer pair 1-2	
Forward primer	GTCCCTATGCACAATGAGTAGCA
Reverse primer	TGGTGCATCCGTTTAGTGGATT
Primer pair 2-1	
Forward primer	GAGGGTCGCGTGCTAATGAT
Reverse primer	AGCGTGACTGCTTACGTGTT
Primer pair 2-2	
Forward primer	ATACTCCTAGGCGAGGGTCG
Reverse primer	AAGCGTGACTGCTTACGTGT

Table S2 Continued

Primers	
MPC2	
Primer pair 1-1	
Forward primer	GGAAGTCATTCCAAAAATGTCCT
Reverse primer	GGAATTGAAGGGTTCACTGACT
Primer pair 1-2	
Forward primer	GGAAGTCATTCCAAAAATGTCCTA
Reverse primer	GGAATTGAAGGGTTCACTGACTA
Pair 2-1	
Forward primer	GCTGAAGTAACTGAACCAAAAGAA
Reverse primer	GGTCCTTTATCTCAGTTGGACA
Pair 2-2	
Forward primer	TAGCTGAAGTAACTGAACCAAAAGA
Reverse primer	TTTGGTCCTTTATCTCAGTTGGAC
Primer pair 3-1	
Forward primer	TTAGTTGACTCGGGCGTGAC
Reverse primer	CCCCTGGAAAACACACTTGG
Primer pair 3-2	
Forward primer	TTTTAGTTGACTCGGGCGTGA
Reverse primer	ACCTTCAGTACTTGGGGGAAC
Primer pair 4-1	
Forward primer	ACGTGAAACCCTACACCACC
Reverse primer	TAAACTGTTTTTGCGGCGCT
Primer pair 4-2	
Forward primer	CGTGAAACCCTACACCACCTT
Reverse primer	TGTAAACTGTTTTTGCGGCG

Table S2 Continued

Primers	
Primer pair 5-1	
Forward primer	CCGACACCTAAACCCTCTGG
Reverse primer	CTTTTCCAGCGCGCTTTCC
Primer pair 5-2	
Forward primer	AGTCCCTGAGGGTGGTCAAC
Reverse primer	CGCTCAGCCGAAGAACCTA
Primers used for the VDAC1 enhancer sequence:	
Primer pair 1	
Forward primer	TTTGAGAGGCATCTGAGGCT
Reverse primer	TGAAGAGAGACTGTGTGGGGA
Primer pair 2	
Forward primer	GGCGAAACAGTGGCATTAGA
Reverse primer	TCATACCTGCCCCCTGTGAC
Primer pair 3	
Forward primer	GCTGTGGGATAGTGCAACCT
Reverse primer	GTACCACTGTGATGCAGCCT
Primer pair 4	
Forward primer	CTCATTCAGGTGCATGTGCG
Reverse primer	GAGTATTTATTTCCCCACCTCCTA
Primer pair 5	
Forward primer	CGAATGGAATGGTCCTTCTGGT
Reverse primer	CATTCCTGGAAGGCTTTGCTTT
Primer pair 6	
Forward primer	AGGTGCAGGAGATGAGGCTTT
Reverse primer	GACTGTACATGGTGTGCTCTG

Table S2 Continued

Primers	
Primer pair 7	
Forward primer	GCAGCAATCCCAAGTAGTCCT
Reverse primer	ACCAGTTTGGCAGCTCTTCA
Primer pair 8	
Forward primer	TCCATGTGAAGAGCTGCCAAA
Reverse primer	ATGGGCCCAACTTTTCACAAAAC

Table S3 Sequences of the regulated genes

VDAC1 and HK1 knock-down plasmids were constructed with the vector Y7823 pLKD-CMV-Puro-U6-shRNA (NC, VDAC1, or HK1)

HK1 KD genes:

HK1 NM_000188 GCCTTTGGAGACGATGGAT

HK1 NM_000188 CCGAGAATGGTGACTTCTT

HK1 NM_000188 GCACCTGCGATGACAGTAT

NC TTCTCCGAACGTGTCACGT

VDAC1 KD genes:

VDAC1 NM_003374 GGATACACTCAGACTCTAA

VDAC1 NM_003374 GGATGGCAAGAACGTCAAT

VDAC1 NM_003374 GGACTGGAATTTCAAGCAT

NC TTCTCCGAACGTGTCACGT

MPC1/2 overexpression:

pLenti-EF1a-EGFP-P2A- blasticidin -CMV-MCS

pLenti-EF1a-EGFP-P2A- blasticidin -CMV-MPC1

pLenti-EF1a-EGFP-P2A- blasticidin -CMV-MPC2

pLenti-EF1a-EGFP-P2A- blasticidin -CMV-MPC1-P2A-MPC2

The sequences are as follows:

MPC1 sequence: NM_016098 CDS

MPC2 sequence: NM_015415 CDS

OPA1 overexpression:

pLenti-CMV-MCS-3FLAG-PGK-Puro and pLenti-CMV-OPA1-PGK-Puro

The OPA1 sequence was NM_015560 CDS

Table S4 Key resource table

Reagent or resource	Source	Identifier
**Chemicals, peptides, and recombinant proteins**		
RPMI-1640 medium	Gibco	C11875500CP
Keratinocyte Serum Free Medium (KSFM)	Gibco	17005042
Cell Counting Kit-8	Dojindo	CK04
Fetal bovine serum (FBS)	Gibco	10099133
Dimethyl sulfoxide (DMSO)	Sigma-Aldrich	276855
Methyl jasmonate	MERCK	39924-52-2
Presto Blue Reagent	Invitrogen	A13261
EdU Cell Proliferation Assay Kit	Ribobio	C10310-1
DAPI (4′,6-diamidino-2-phenylindole, dihydrochloride)	Invitrogen	D1306
Crystal violet staining solution	Sangon Biotech	E607309-0100
Cell Cycle Staining Kit	MultiSciences	CCS012
Annexin V-FITC/PI apoptosis kit	Multiscience	AP101
UK5099	MCE	PF-1005023
Luciferase-based ATP Assay Kit	Beyotime	S0026
$^{13}C_6$-glucose(D-GLUCOSE(1,2-13C2,99%))	Cambridge Isotope Laboratories	CLM-504-PK
JC-1	Thermo Scientific	M34152
Calcium Detection Kit	Bestbio	BB-48112-2
Pierce BCA Protein Assay Kit	Thermo Scientific	23225
TRIzol reagent	Invitrogen	15596026
Prime Script RT Reagent Kit	TaKaRa	RR037B
SYBR Green	TaKaRa	RR820B
Cell Mitochondria Isolation Kit	Beyotime	C3601
CellTiter-Blue Cell Viability Assay	Promega	G8080
Simple ChIP Enzymatic Chromatin IP Kit (Magnetic Beads)	CST	9003
Lipofectamine 3000 Reagent	Thermo Scientific	L3000001
2-Solution DAB Kit	Invitrogen	882014
Blasticidin	Selleck	S7419
Janus Green B	Solarbio	J8020
**Antibodies**		
Anti-MPC1	CST	14462
Anti-MPC2	CST	46141
Anti-MPC2	Proteintech	20049-1-AP
Anti-MFN1	Proteintech	13798-1-AP
Anti-MFN2	Proteintech	12186-1-AP

Table S4 Continued

Reagent or resource	Source	Identifier
Anti-OPA1	Proteintech	27733-1-AP
Anti-DRP1	Proteintech	12957-1-AP
Anti-cytochrome C	Proteintech	10993-1-AP
Anti-VDAC1/Porin	Proteintech	55259-1-AP
Anti-OMA1	Proteintech	17116-1-AP
YME1L1	Proteintech	11510-1-AP
Glycolysis Antibody Sampler Kit	CST	8337
Anti-histone H3 (acetyl K27), ChIP grade	Abcam	ab4729
Anti-β-Actin (13E5) rabbit mAb	CST	4970S
Anti-β-Tubulin	CST	2146
Anti-AR	CST	5153
**Experimental Models**		
Nude mice	Shanghai Laboratory Animal Center, SLAC, China	
NOD-SCID mice	Shanghai Laboratory Animal Center, SLAC, China	
**Experimental Equipment**		**Version**
Microplate reader	Molecular Devices	Paradigm
Microplate reader	Tecan	M200PRO
Nano Zoomer	Hamamatsu	S60
Microscope	Leica	OMI4000B
Microscope	ZEISS	Observer. D1
NanoDrop	Thermo	2000c
Seahorse XF96	Agilent	
Irradiation Center	Faculty of Naval Medicine, Second Military Medical University	
Real-time PCR system	Thermo	QuantStudio 7
GCMS-QP	Shimadzu	2010 Plus quadrupole mass analyzer
^{18}F-FDG micro-PET/CT imaging	Super Nova	
**Experimental Software**		**Version**
Microplate reader software	Molecular Devices	SoftMax Pro 6.3
Flow cytometer	MACSQuant	Analyzer 10
Nano Zoomer	Hamamatsu	NDP.scan 3.2
Avatar (PET-CT) software	Pingseng	1.2
SPSS	SPSS Inc.	19.0
GraphPad		Prism 5
Wave	Agilent	2.6.0.31

Table S4 Continued

Reagent or resource	Source	Identifier
Leica LAS X	Leica	LAS X
FlowJo		7.6
ImageJ		1.46 r

Table S5 Patient characteristics in metabolic analysis

	PCa (discovery set)	PCa	BPH
Number	25	51	19
Age (year)	69.0 ± 7.2	68.3 ± 7.4	65.2 ± 11.8
PSA (ng/mL)	21.7 ± 21.3	23.2 ± 18.7	7.7 ± 11.5
GS GS<7	2	6	/
GS=7	12	31	/
GS>7	11	14	/

GS, Gleason score.

Table S6 Patient characteristics in group A and group B

	Group A	Group B
Number	65	125
Age (year)	68.8 ± 10.8	68.6 ± 6.4
PSA (ng/mL)	27.8 ± 37.6	41.6 ± 110.6
GS	7.5 ± 1.0	7.7 ± 1.0

GS, Gleason score.
